# Wnt activation promotes memory T cell polyfunctionality via epigenetic regulator PRMT1

**DOI:** 10.1172/JCI140508

**Published:** 2022-01-18

**Authors:** Bo-Yi Sung, Yi-Hsin Lin, Qiongman Kong, Pali D. Shah, Joan Glick Bieler, Scott Palmer, Kent J. Weinhold, Hong-Ru Chang, Hailiang Huang, Robin K. Avery, Jonathan Schneck, Yen-Ling Chiu

**Affiliations:** 1 Institute of Cell Engineering and; 2 Department of Pathology, School of Medicine, Johns Hopkins University, Baltimore, Maryland, USA.; 3 Department of Microbiology and Immunology,; 4 Department of Biomedical Engineering, and; 5 Department of Obstetrics and Gynecology, Tri-Service General Hospital, National Defense Medical Center, Taipei, Taiwan.; 6 Division of Pulmonary and Critical Care Medicine, Department of Medicine, School of Medicine, Johns Hopkins University, Baltimore, Maryland, USA.; 7 Department of Medicine and; 8 Department of Surgery, and Department of Immunology, Duke University Medical Center, Durham, North Carolina, USA.; 9 Analytic and Translational Genetics Unit, Massachusetts General Hospital, Boston, Massachusetts, USA.; 10 Division of Infectious Diseases, Department of Medicine, School of Medicine, Johns Hopkins University, Baltimore, Maryland, USA.; 11 Department of Medicine and Oncology, School of Medicine, Johns Hopkins University, Baltimore, Maryland. USA.; 12 Graduate Institute of Medicine and Graduate Program in Biomedical Informatics, Yuan Ze University, Taoyuan, Taiwan.; 13 Department of Medical Research, Far Eastern Memorial Hospital, Taipei, Taiwan.; 14 Graduate Institute of Clinical Medicine, National Taiwan University College of Medicine, Taipei, Taiwan.

**Keywords:** Immunology, Adaptive immunity, Epigenetics, T cells

## Abstract

T cell polyfunctionality is a hallmark of protective immunity against pathogens and cancer, yet the molecular mechanism governing it remains mostly elusive. We found that canonical Wnt agonists inhibited human memory CD8^+^ T cell differentiation while simultaneously promoting the generation of highly polyfunctional cells. Downstream effects of Wnt activation persisted after removal of the drug, and T cells remained polyfunctional following subsequent cell division, indicating the effect is epigenetically regulated. Wnt activation induced a gene expression pattern that is enriched with stem cell–specific gene signatures and upregulation of protein arginine methyltransferase 1 (PRMT1), a known epigenetic regulator. PRMT1^+^CD8^+^ T cells are associated with enhanced polyfunctionality, especially the ability to produce IL-2. In contrast, inhibition of PRMT1 ameliorated the effects of Wnt on polyfunctionality. Chromatin immunoprecipitation revealed that H4R3me2a, a permissive transcription marker mediated by PRMT1, increased at the IL-2 promoter loci following Wnt activation. In vivo, Wnt-treated T cells exhibited superior polyfunctionality and persistence. When applied to cytomegalovirus (CMV) donor–seropositive, recipient-seronegative patients (D+/R–) lung transplant patient samples, Wnt activation enhanced CMV-specific T cell polyfunctionality, which is important in controlling CMV diseases. These findings reveal a molecular mechanism governing T cell polyfunctionality and identify PRMT1 as a potential target for T cell immunotherapy.

## Graphical abstract

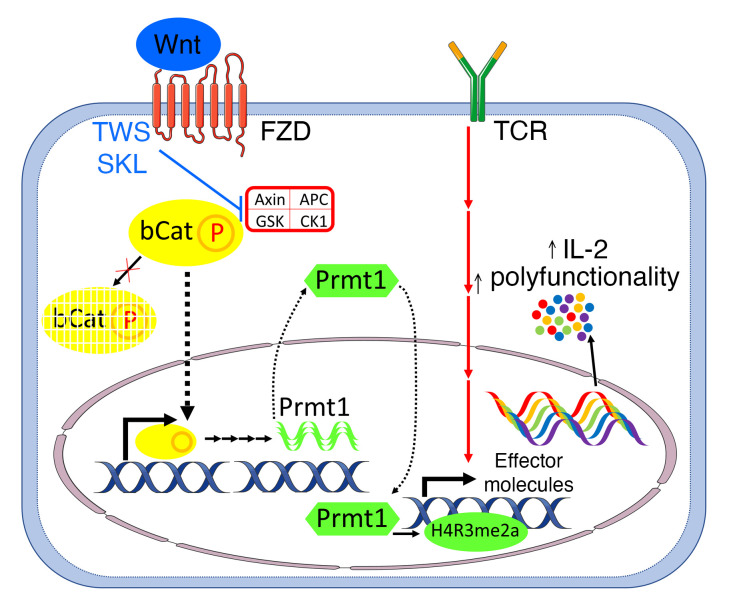

## Introduction

After antigen encounter, naive T cells (TN cells) differentiate into memory T cells that persist for decades via homeostatic proliferation and continuously maintain their ability to respond to the same antigen upon secondary exposure (1, 2). In humans, the composition of the memory T cell pool is highly heterogeneous, containing a diverse array of phenotypic and functional subsets (3). Among them, polyfunctional T cells capable of simultaneously producing multiple effector molecules have been shown to provide superior protective immunity in various human and murine infectious diseases (4–6) and cancer (7). When chronically stimulated with a high level of antigen, T cells gradually lose the ability to produce IL-2, TNF-α, and IFN-γ in a hierarchical manner and thus their polyfunctional phenotype (8, 9). T cell exhaustion is thus defined as the terminal stage of T cells when they eventually lose all effector functions due to strong and persistent antigen stimulation (4, 5, 10). The exhaustion state is both controlled by the balance of transcription factors T-bet and Eomes and also characterized by the upregulation of inhibitory receptors (11), such as programmed cell death protein 1 (PD-1) and cytotoxic T lymphocyte–associated antigen 4 (CTLA-4) (12, 13). Despite remarkable successes using checkpoint blockade antibodies to treat metastatic cancer (12, 14), only a portion of patients benefit from this type of treatment (15, 16); this is partially due to the fact that the molecular mechanisms governing T cell polyfunctionality are not fully understood.

The Wnt signaling pathway controls cell fate in a variety of cell types, even beyond the embryonic stem cell stage (17, 18). For example, it has been reported that Wnt signaling is critical for thymocyte differentiation and survival (19). During canonical Wnt pathway activation, Wnt proteins bind to Frizzled (Fzd), preventing the proteasomal degradation of β-catenin, allowing β-catenin translocation to the nucleus to mediate gene transcription along with T cell factor/lymphocyte enhancer factor (Tcf/lef). In the absence of Wnt proteins, β-catenin is phosphorylated by the destruction complex composed of adenomatosis polyposis coli (APC), Axin, casein kinase 1 (CK1), and glycogen-synthase kinase 3β (GSK-3β) (17). Small molecules have been developed to activate the canonical Wnt signaling pathway by suppressing the formation of the destruction complex and effecting mature T cell differentiation and function. Gattinoni et al. demonstrated that TWS119, a GSK-3β inhibitor, promotes the generation of stem cell memory T cells (TSCM cells) from naive T cells (20). Another study also demonstrated that activation of the Wnt pathway in TN cells triggers robust and long-lasting memory T cell formation (21). However, whether Wnt signaling affects antigen-experienced T cells’ polyfunctionality was not clear.

Cytomegalovirus (CMV) is a chronic infection that is known to continuously promote the accumulation of dysfunctional and terminally differentiated memory T cells (22, 23). In recent years, there have been major improvements in the prevention, diagnosis, and treatment of CMV-associated diseases that have subsequently reduced morbidity and mortality rates. However, recurrent episodes of CMV viremia are still associated with graft failure and even death in solid organ transplant and hematopoietic stem cell transplant recipients (24–26). Among the 4 serostatus groups in lung transplant (LT) recipients, CMV donor–seropositive, recipient-seronegative (D+/R–) patients are the most vulnerable to CMV disease (27), since the transplant recipient acquires CMV via donor-derived transmission without antecedent CMV-specific immunity in the recipient. A recent review outlines the importance of virus-specific T cells in controlling viral replication in transplant recipients (28). CMV-specific T cell polyfunctionality profile is also a critical factor in controlling CMV disease in mismatched D+/R– LT patients. Among all CMV-reactive T cells, CD8^+^ T cells that produce only IFN-γ are the most abundant subtype, but they most likely represent nonprotective memory cells. In contrast, dual IFN-γ/IL-2–producing CD8^+^ T cells are present in patients controlling their CMV reactivations, but absent from noncontrollers (those who are unable to prevent recurrent reactivation episodes of CMV; refs. 29, 30).

In the current study, we discovered that both TWS119 and SKL2001, small molecules activating the canonical Wnt pathway, inhibit human memory T cell differentiation into effector cells. Unexpectedly, Wnt activation also enhanced T cell polyfunctionality, as indicated by the coexpression of IFN-γ, TNF-α, IL-2, and CD107a. This improvement of T cell functionality was sustained even after removal of the Wnt agonists and is independent of signals from DCs. We hypothesize that the effects of Wnt activation on memory T cells are epigenetically regulated. Gene set enrichment analysis (GSEA) revealed that the gene expression profile of Wnt-activated memory CD8^+^ T cells was enriched with stem cell–related genes. We further identified the improvement in T cell polyfunctionality as associated with the upregulation of protein arginine methyltransferase 1 (PRMT1), an enzyme that catalyzes histone arginine methylation. Confirming this observation, shRNA-mediated repression of PRMT1 abrogated T cell polyfunctionality and reversed the effects of Wnt activation. ChIP revealed that the chromatin structure at the IL-2 locus was remodeled as H4R3me2a, a permissive histone marker, mediated by PRMT1. Wnt-activated T cells demonstrated superior survival, homeostatic maintenance, and antiapoptotic capabilities in vivo and enhanced CMV-specific polyfunctionality in CMV D+/R– LT patients. These findings broaden our understanding of molecular mechanisms that control memory T cell polyfunctionality and highlight potential therapeutic pathways for improving T cell function.

## Results

### Wnt signaling alters human memory CD8^+^ T cell proliferation and differentiation.

**Figure 1:**
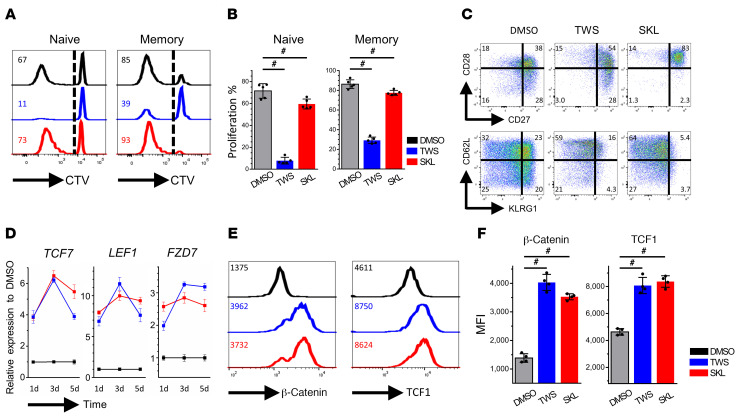
Wnt signaling alters human memory CD8^+^ T cell proliferation and differentiation. (**A**) FACS-sorted human naive and memory CD8^+^ T cells were stained with CTV and stimulated with CD3/CD28 in DMSO (black), TWS119 (blue), and SKL2001 (red) for 7 days. Numbers represent the percentages of divided cells in each treatment. (**B**) Average proliferative response for each treatment (*n =* 5). Dunn’s test for multiple comparisons. (**C**) Memory CD8^+^ T cells in different treatments for 7 days were stained with phenotypic markers. Numbers represent the percentages of cells in each quadrant. (**D**) Quantitative RT-PCR analysis of Tcf7, Lef1, and Fzd7 in memory CD8^+^ T cells with or without Wnt agonist treatment on days 1, 3, and 5. Numbers are normalized to marker expression of the DMSO group at each corresponding time point. (**E**) Memory CD8^+^ T cells were stimulated with CD3/CD28 and treated with or without Wnt agonists for 7 days. β-Catenin and TCF1 levels were analyzed by flow cytometry. Numbers represent the MFI of each protein. (**F**) Average MFI of β-catenin and TCF1 in different treatment conditions (*n =* 4). Dunn’s test for multiple comparisons. ^#^*P* < 0.05.

TWS119 is a small molecule Wnt agonist known to induce TSCM cells from TN cells in mice (20). In humans, TSCM cells are also identified and able to differentiate into multiple subtypes of memory T cells, including central memory (TCM), effector memory, and effector T cells, with an improved proliferative capacity (31). A major obstacle to TWS119 application in vivo is that TWS119 severely inhibits cell proliferation and likely triggers many off-target pathways in addition to Wnt due to its suppression of GSK3 function, which is involved in many signaling cascades (17). Identified in 2012, another small molecule, SKL2001, specifically disrupts the interaction between Axin and β-catenin (32), providing a more targeted activation of the canonical Wnt pathway. We hypothesized that SKL2001 also affects human memory T cell differentiation and function. As shown in Figure 1, FACS-sorted human naive (CD8^+^, CD45RA^hi^CCR7^hi^) and memory (CD8^+^, non-CD45RA^hi^CCR7^hi^) CD8^+^ T cells from healthy donors were stimulated with anti-CD3/CD28 antibodies in the presence or absence of Wnt agonists for 7 days. In a CellTrace Violet (CTV) dilution proliferation assay, TWS119 intensively inhibited cell proliferation, with only 39% of cells dividing, whereas SKL2001 treatment resulted in only mild proliferation suppression compared with that of untreated cells in both naive and memory T cell populations (Figure 1, A and B). We further analyzed T cell differentiation markers and costimulatory molecules that reflected the status and quality of CD8^+^ T cells. Wnt agonists increased the expression of CD27, CD28, and CD62L, whereas the expression levels of the senescent marker KLRG1 were downregulated (Figure 1C). The results indicate that Wnt agonists generate more TCM cells phenotypically due to the increase in CD62L and decrease in KLRG1 expression.

To verify the effects of TWS119 and SKL2001 on the Wnt pathway, mRNA levels of TCF7, LEF1, and FZD7 were measured by quantitative reverse-transcriptase PCR (RT-PCR) 1, 3, and 5 days after ex vivo stimulation. At each time point, expression levels of Wnt-related genes were at least 2-fold greater in cells treated with either TWS119 or SKL2001 than in untreated cells (Figure 1D). The MFIs of β-catenin and TCF1, the major canonical Wnt-responding molecules, were higher in the Wnt agonist–treated groups, as measured by flow cytometry (Figure 1, E and F). The results demonstrate that both TWS119 and SKL2001 treatment can generate high-quality T cells through the upregulation of the Wnt pathway and that SKL2001 has a significantly less inhibitory effect on T cell proliferation.

### Wnt signaling enhances memory CD8^+^ T cell polyfunctionality independently of proliferation.

**Figure 2:**
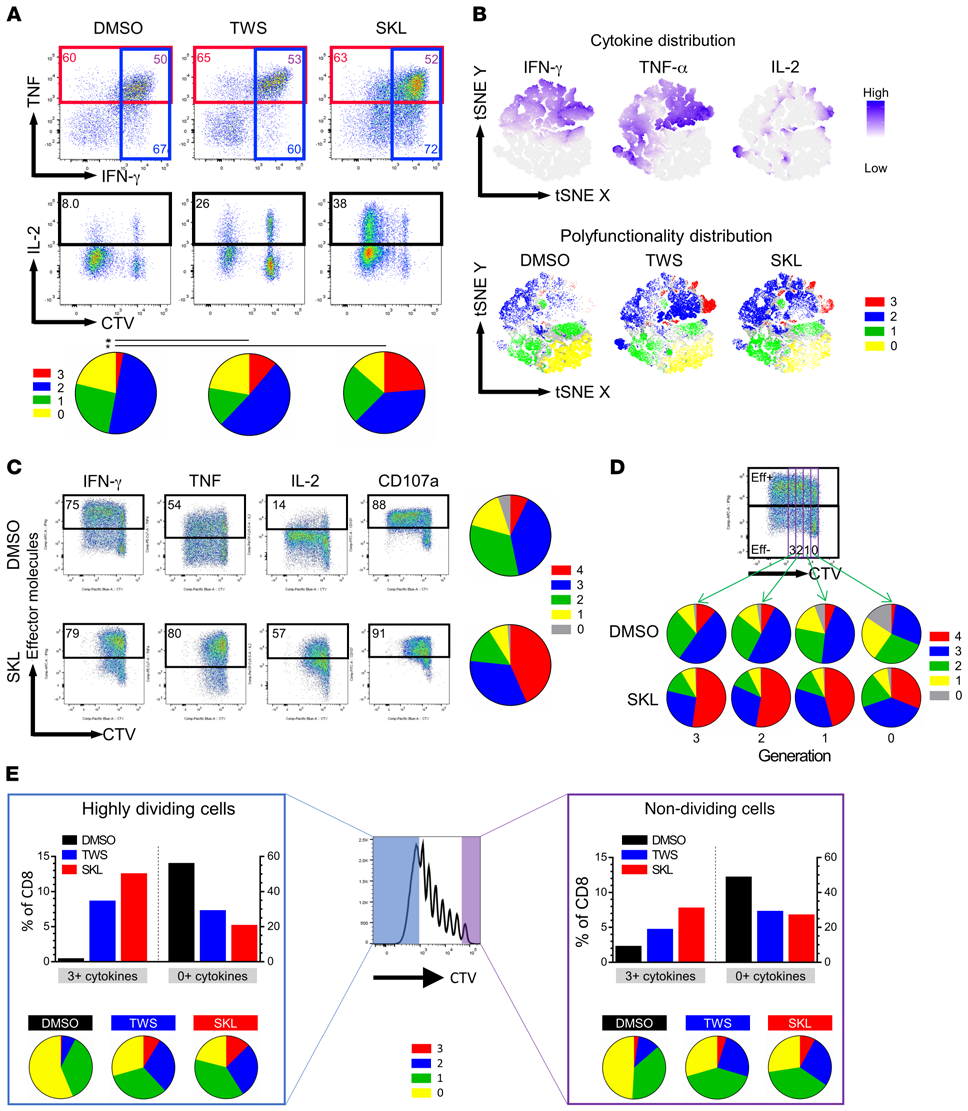
Wnt signaling enhances memory CD8^+^ T cell polyfunctionality independently of cell proliferation. (**A**) Memory CD8^+^ T cells stained with CTV on day 0 were stimulated with CD3/CD28 for 7 days in different conditions (*n =* 5) and analyzed for polyfunctionality by PMA/ionomycin stimulation. Representative intracellular cytokine staining for the polyfunctionality assay is shown. Top: TNF-α^+^ population (red) and IFN-γ^+^ population (blue). Purple numbers indicate the double-positive percentage (right upper quadrant [RUQ]). Bottom: staining for CTV versus IL-2. Pie charts summarize the polyfunctionality assay in different treatment conditions. Red slices indicate proportion of cells positive for all 3 cytokines (IFN-γ^+^, TNF-α^+^, and IL-2^+^). *P < 0.05, **P < 0.01, ***P < 0.001. 0, 1, 2, 3, 4 are defined as the number of positive cytokine. (**B**) Memory CD8^+^ T cell cytokine production from 5 donors treated with 3 conditions were detected by flow cytometer; 10,000 cells from each sample were combined to run tSNE distribution. After the tSNE *x* and tSNE *y* coordinate of each cell was determined, cells were labeled based on the MFI of 3 different cytokines (top) or polyfunctionality (bottom). (**C**) Memory CD8^+^ T cells stimulated with CD3/CD28 were analyzed for polyfunctionality in early proliferation generations on day 4. IFN-γ, TNF-α, IL-2, and CD107a were assessed. Pie charts show the summary of the polyfunctionality assay with each treatment. (**D**) Numbers in purple boxes indicate the different generations of CD8^+^ T cells. Polyfunctionality of different generations in DMSO and SKL2001 treatment were computed and presented by pie charts. (**E**) Memory CD8^+^ T cells stained with CTV were stimulated with CD3/CD28 for 7 days. On day 7, cells were sorted into highly divided and nondivided groups based on their CTV intensity. After sorting, 6 samples (DMSO/TWS119/SKL2001 by CTV_hi/lo_) were further stimulated with CD3/CD28 without any Wnt pathway modulation for another 7 days before being analyzed for polyfunctionality.

To assess how the Wnt pathway affects memory T cell function, FACS-sorted memory CD8^+^ cells were cultured for 7 days, and cells were analyzed for multiple cytokines and proliferation by flow cytometry. Wnt agonists did not increase the size of IFN-γ^+^ and TNF-α^+^ populations, whereas the percentages of IL-2^+^ cells were dramatically higher in TWS119 and SKL2001 treatment (7% vs. 28% and 42%) (Figure 2A and Supplemental Figure 1; supplemental material available online with this article; https://doi.org/10.1172/JCI140508DS1). The degree of polyfunctionality is represented by pie charts in which red slices labeled with 3 indicate the proportion of the entire CD8^+^ cell population positive for 3 effector molecules, namely IFN-γ, TNF-α, and IL-2 (Figure 2A). Following Wnt agonist stimulation, the MFIs of IFN-γ, TNF-α, and IL-2 were increased, demonstrating that cells were capable of producing more effector molecules per cell (Supplemental Figure 1). To analyze polyfunctionality from multiple donors and treatments, we used tSNE (t-distributed stochastic neighbor embedding) to incorporate all samples. CD8^+^ T cells from 5 donors and 3 treatments were acquired by flow cytometer, and each cell was clustered according to the similarity of its cytokine production pattern. TWS119 and SKL2001 induced more polyfunctional cells (red and blue), whereas DMSO rendered cells dysfunctional (yellow and green; Figure 2B). Both TWS119 and SKL2001 enhanced polyfunctionality in memory CD8^+^ cells, whereas TWS119 suppressed T cell function in naive CD8^+^ cells (Supplemental Figure 2). Notably, naive and memory T cells are traditionally categorized by CD45RA/CCR7 or CD45RA/CD62L expression. Here, we compared the CCR7- and CD62L-defined naive and memory T cells and found a high degree of correlation phenotypically and functionally (Supplemental Figure 3).

Wnt agonists suppressed cell proliferation in CD8^+^ memory T cells; thus, we could not exclude the possibility that the superior polyfunctionality we observed was due to the inhibition of proliferation. To address this, we stained FACS-sorted memory CD8^+^ T cells with CTV and stimulated them for 4 days to analyze the polyfunctionality of individual generations in the presence or absence of SKL2001. Based on the intensity of CTV, we identified early T cell generations (≤3 divisions) and assessed their cytokine production. IFN-γ, TNF-α, and IL-2 were all increased, and polyfunctionality was enhanced across different cell generations in the presence of SKL2001. The degree of polyfunctionality within each treatment positively correlated with cell division, which may reflect the intensity of stimulation (Figure 2, C and D). However, the percentage of cells positive for CD107a, also known as lysosomal-associated membrane protein 1 (LAMP-1), a degranulation marker, was not upregulated by SKL2001. These results indicate that SKL2001 increases memory CD8^+^ T cell polyfunctionality independently of cell proliferation.

We further investigated whether Wnt signaling can prevent the loss of polyfunctionality in extensively proliferated CD8^+^ T cells and sustain polyfunctionality even after removal of the Wnt agonists. To explore this, we sorted nondivided (CTV_hi_) and highly divided (CTV_lo_) CD8^+^ cells after 7 days of stimulation in the presence or absence of Wnt agonists and further stimulated cells for another week without Wnt activation. On day 14, polyfunctionality was analyzed and compared. Comparing CTV_lo_ cells with CTV_hi_ cells in the DMSO group, lower polyfunctionality was observed in the CTV_lo_ group (Figure 2E). In contrast, CTV_lo_ cells demonstrated a more robust polyfunctional response than CTV_hi_ cells in both the TWS119- and SKL2001-treated groups (Figure 2E). Both the CTV_lo_ and CTV_hi_ populations from previously Wnt-activated culture were also able to maintain superior polyfunctionality compared with those in DMSO-treated groups; for example, 9% and 13% of CTV_lo_ cells expressed 3 positive cytokines in the TWS119- and SKL2001-treated compared with just 1% in the DMSO-treated group (Figure 2E). The fold change of cell number among CTV_hi_ and CTV_lo_ groups was comparable, indicating that T cells underwent an equivalent degree of proliferation during the second round of stimulation. Collectively, these results show that Wnt signaling elicits improved T cell polyfunctionality independently of cell proliferation and that the beneficial effects on polyfunctionality can be preserved and demonstrated after restimulation.

### Wnt agonists increase antigen-specific CD8^+^ T cell polyfunctionality independently of APC stimulation and checkpoint blockade.

**Figure 3:**
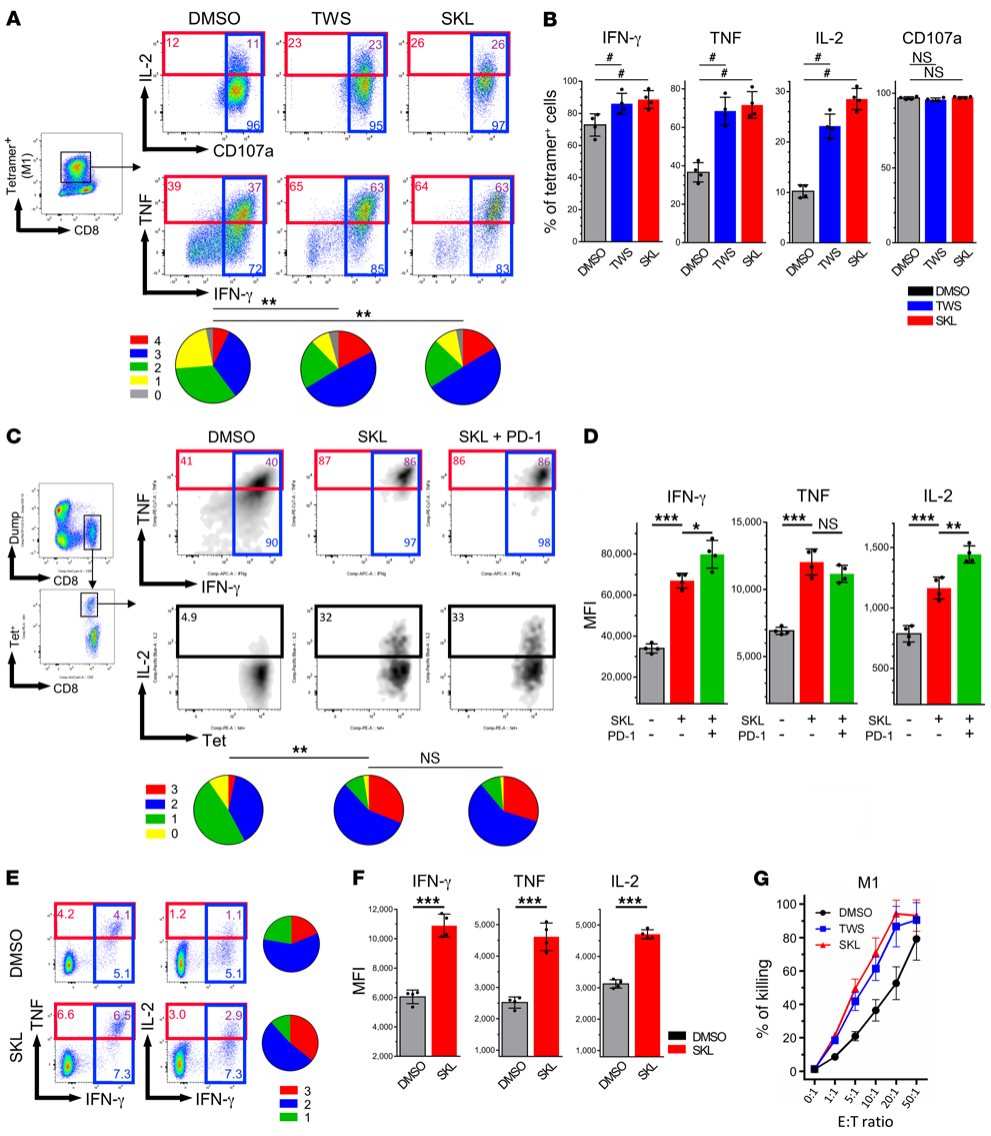
Wnt activation enhances CD8^+^ T cell polyfunctionality independently of inhibitory receptor and DC signaling. (**A**) CD8^+^ T cells from HLA-A*0201 donors were stimulated with autologous moDCs pulsed with influenza M1 peptide for 7 days (*n =* 4). Tetramer-positive cells were subsequently assessed for polyfunctionality by restimulation with M1-pulsed HLA-A*0201^+^ T2 cells. Blue, red, and black boxes and numbers indicate IFN-γ^+^, TNF-α^+^, and IL-2^+^ cells, respectively. Purple numbers indicate the percentages of IFN-γ^+^TNF-α^+^ double-positive cells. Pie charts represent the T cell polyfunctionality profile induced by different treatments. Red slices indicate the proportion of cells positive for four effector functions (IFN-γ^+^, TNF-α^+^, IL-2^+^, and CD107a^+^). 0, 1, 2, 3, 4 are defined as the number of positive cytokine. (**B**) Percentages of IFN-γ–, TNF-α–, IL-2-, and CD107a-positive cells among M1-specific cells (*n =* 4). Dunn’s test for multiple comparisons. (**C**) Peripheral blood mononuclear cells from HLA-A*0201 donors were collected and stimulated with M1 peptide for 7 days in DMSO and isotype control antibodies or in SKL2001 with PD-1 blockade antibodies or isotype control antibodies. Cells were harvested and further stimulated by M1-pulsed T2 cells to analyze polyfunctionality (*n =* 4). (**D**) MFI of IFN-γ, TNF-α, and IL-2 in corresponding cytokine-positive cells in different treatment conditions (*n =* 4). Mann-Whitney *U* test. (**E**) HLA-A*0201 CD8^+^ T cells were stimulated with M1-pulsed aAPCs for 7 days in different treatment conditions and analyzed for polyfunctionality. (**F**) MFI of individual cytokines in corresponding cytokine-positive cells (*n =* 4). Mann-Whitney *U* test. (**G**) Cytotoxicity assay showed enhanced cytotoxicity in Wnt agonist–treated M1-specific T cells. *P < 0.05; **P < 0.01; ***P < 0.001. ^#^*P* < 0.05 by Dunn’s test.

The previous experiments were all performed in memory T cells. We further investigated the effects of Wnt agonists on antigen-specific CD8^+^ T cells’ polyfunctionality induced by autologous monocyte-derived DCs (moDCs). HLA-A*0201–restricted immunodominant influenza peptide M1_58–66_ (GILGFVFTL) was loaded on autologous moDCs to induce the expansion of M1-specific CD8^+^ T cells from HLA-A*0201^+^ donors with or without Wnt agonists. In accordance with our previous findings (5), a distinct population of M1-specific polyfunctional T cells can be generated on day 7. We observed that Wnt agonists increased the percentages of IFN-γ^+^, TNF-α^+^, and IL-2^+^ cells, but not CD107a^+^ cells (Figure 3, A and B). The 4-function polyfunctional cell response in M1-specific CD8^+^ T cells was also enhanced (Figure 3A). The enhancement of polyfunctionality was also observed in other moDC-based antigen-specific stimulation (Supplemental Figure 4).

Next, we studied the relationship between Wnt activation and inhibitory receptor signaling on T cell polyfunctionality, since several studies have shown that checkpoint blockades, including anti–PD-1 and anti–CTLA-4 therapy, restore the polyfunctionality of exhausted T cells (33). We found that SKL2001 significantly increased the proportion of polyfunctional cells, from 4% to 31%, but surprisingly, anti–PD-1 antibodies did not further enhance T cell polyfunctionality in terms of cytokine-positive population percentages (Figure 3C). However, PD-1 blockade elevated the MFI of IFN-γ and IL-2 in Wnt-activated cells (Figure 3D), indicating that, whereas Wnt pathway activation improved polyfunctionality, PD-1 blockade further increased the amount of cytokines produced per cell.

To eliminate the potential impact of Wnt stimulation on DCs, we utilized artificial antigen-presenting cells (aAPCs) that contained HLA-A2–Ig-M1 complex (signal 1) and anti-CD28 (signal 2) (34) to induce M1-specific cell expansion. The results showed that SKL2001 increased the polyfunctionality and MFI of cytokines in an aAPC stimulation experiment (Figure 3, E and F), which indicated that the improved polyfunctional responses are not dependent on DCs. Finally, M1-specific CD8^+^ T cells were cocultured with M1-pulsed HLA*0201–positive T2 cells (target cells) in various ratios for 6 hours to directly evaluate T cell cytotoxicity. Wnt-activated M1-specific cells had increased antigen-specific cytolytic function (Figure 3G). Overall, our results demonstrate that enhancement of T cell polyfunctionality is a result of increased cytokine production through Wnt activation and is T cell intrinsic.

### Microarray analysis revealed unique molecular signatures associated with T cell polyfunctionality and stem-like properties.

**Figure 4:**
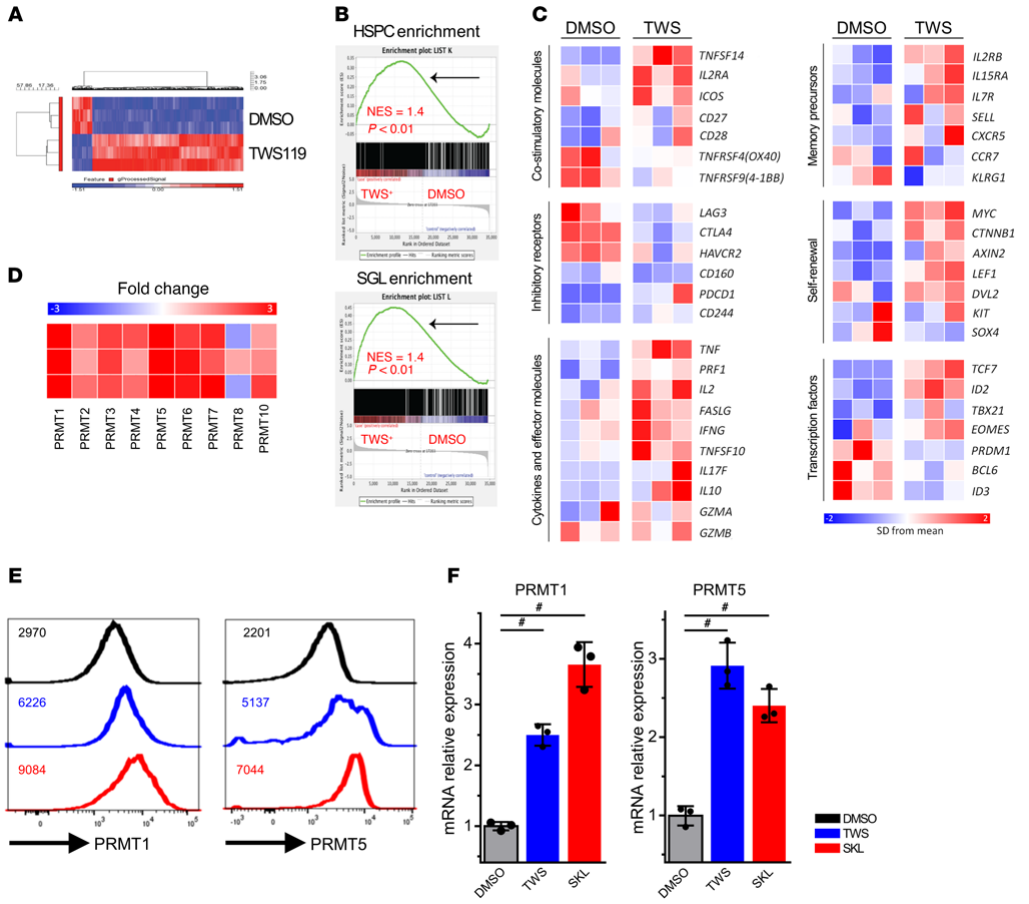
Polyfunctional T cells are enriched with stem cell–associated genetic signatures. (**A**) Heatmap of gene expression of memory CD8^+^ T cells treated by DMSO or TWS119 for 7 days. Red and blue reflect increased and decreased gene expression, respectively. (**B**) GSEA demonstrated that TWS119-treated memory T cells were enriched with progenitor and hematopoietic stem cell signatures. (**C**) Heatmap showing the expression of immunological- and self-renewal–related genes. (**D**) Fold change of PRMT gene expression following TWS119 treatment. (**E**) PRMT1 and PRMT5 expression in memory CD8^+^ T cells was upregulated by Wnt agonist treatment. Numbers indicate MFI value of each protein. (**F**) Quantitative RT-PCR analysis of PRMT1 and PRMT5 in response to Wnt activation (*n =* 3). Dunn’s test for multiple comparisons. ^#^*P* < 0.05 by Dunn’s test.

We hypothesized that there exists a unique molecular mechanism for the upregulation of T cell polyfunctionality mediated by Wnt pathway activation. To identify the transcriptomic signature of memory T cell polyfunctionality, FACS-sorted human memory CD8^+^ cells were stimulated in the presence of TWS119 for 7 days before microarray gene expression analysis. A heatmap of gene expression showed that 124 genes were downregulated and 927 genes were upregulated following Wnt treatment, with the threshold setting of more than 1.5-fold change and *P* values of less than 0.05 (Figure 4A). GSEA demonstrated that Wnt-treated cells were significantly enriched with stemness and Wnt-related gene signature (Figure 4B and Supplemental Figure 5D). We further examined several gene clusters, including coreceptors, cytokines, naive/memory surface markers, self-renewal genes, and transcription factors, and found that Wnt-treated memory cells contained characteristics of TCM cells (Figure 4C).

To verify the microarray results, we analyzed the effects of TWS119 and SKL2001 treatment by RNA-Seq analysis. RNA-Seq showed that Wnt agonists induced stem cell–related genes, yet suppressed exhaustion-related genes (Supplemental Figure 5A). GSEA was exploited to compute the similarity of transcriptomic profiles between TWS119 and SKL2001. TWS119-regulated genes were extracted and applied to SKL2001-treated samples and vice versa. The GSEA analysis showed that TWS119 and SKL2001 induced a common global transcriptomic signature. (Supplemental Figure 5C). We used Gene Ontology (GO) analysis on the Database for Annotation, Visualization, and Integrated Discovery (DAVID) platform. In our transcriptomic data, we found that most members in the enzymatic protein family PRMTs were upregulated (Figure 4D and Supplemental Figure 5B) after Wnt activation. Previous studies have shown that the PRMTs control cell stemness and differentiation in many tissues (35). Hence, we focused on PRMTs’ regulation of T cell stemness and functionality.

PRMTs catalyzing protein methylation at arginine residues play an important role in diverse cellular processes, including transcription regulation, RNA processing, and signaling pathways, in several pathological diseases, including tumorigenesis, cardiovascular diseases, and autoimmune diseases (36, 37). Among all the PRMTs, PRMT1 and PRMT5 are the major enzymes responsible for asymmetric dimethylarginine (aDMA, catalyzed by type I members) and symmetric dimethylarginine (sDMA, catalyzed by type II members) modifications, respectively (36). To validate the role of PRMTs in T cell polyfunctionality, CD8^+^ T cells treated with or without Wnt agonists were harvested to investigate the protein and mRNA expression levels of PRMT1 and PRMT5. Both PRMT1 and PRMT5 protein and mRNA levels were upregulated in the presence of Wnt agonists, as detected by flow cytometry and quantitative RT-PCR (Figure 4, E and F). Due to the fact that the number of PRMT5 variants was greater than that of PRMT1, we chose to focus on PRMT1 in the following studies to circumvent the complexity and uncertainty caused by the dynamics of protein variants.

### PRMT1 highly correlates with T cell polyfunctionality.

**Figure 5:**
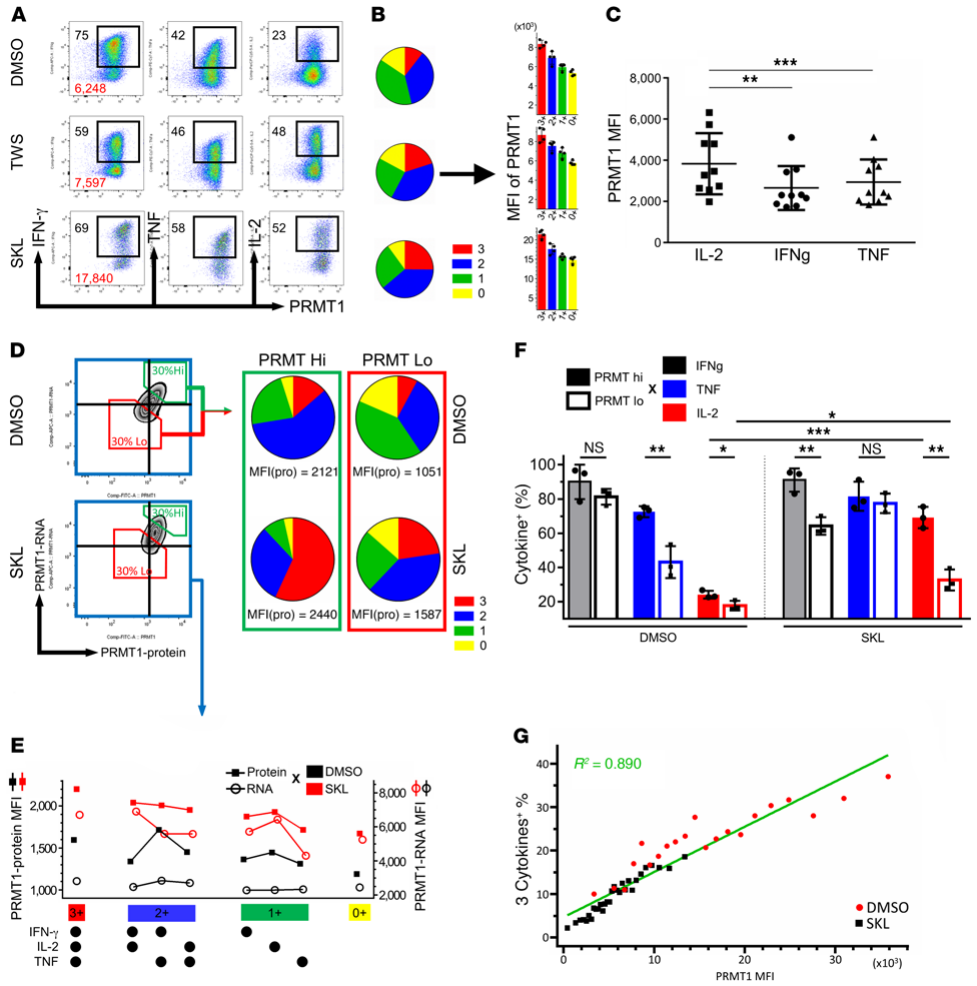
PRMT1 highly correlates with CD8^+^ T cell polyfunctionality. (**A**) Intracellular staining for cytokines and PRMT1 expression in memory CD8^+^ T cells in different treatment conditions. PRMT1 staining is illustrated on the *x* axes versus cytokine production (*y* axes). Black and red numbers represent percentages of cytokine-positive cells and PRMT1 MFI, respectively. (**B**) Pie charts showing the polyfunctionality profile of the different treatment conditions. Bar graphs display PRMT1 MFI of T cell subsets of different degrees of polyfunctionality of each treatment condition (*n =* 4). 0, 1, 2, 3 are defined as the number of positive cytokines. (**C**) PRMT1 MFI in IL-2^+^, IFN-γ^+^, or TNF-α^+^ CD8^+^ cells undergoing 7 days of CD3/CD28 stimulation from multiple healthy donors (*n =* 10). Mann-Whitney *U* test. (**D**) Combined PRMT1 protein and RNA detection with polyfunctionality profile by flow cytometry. Green and red boxes represent top and bottom 30% of PRMT1-expressing cells, respectively. Black lines in the plots indicate the MFI of PRMT1 protein and RNA in control group. Pie charts show the polyfunctionality profile of PRMT1_hi/lo_ (green/red) cells in the presence or absence of SKL2001. (**E**) MFI of PRMT1 protein and mRNA were plotted by different polyfunctionality combinations. Squares and circles represent PRMT1 protein and mRNA levels, respectively. (**F**) Percentages of IFN-γ^+^ (black), TNF-α^+^ (blue), and IL-2^+^ (red) of all CD8^+^ cells in PRMT1 hi versus lo cells (filled or grid) following treatment with DMSO or SKL2001 (*n =* 3). Mann-Whitney *U* test. (**G**) Memory CD8^+^ cells treated with DMSO or SKL2001 were stratified into approximately 20–30 tiers according to MFI of PRMT1. Each tier contained at least 500 cells, and polyfunctionality was computed from individual tiers before being plotted against PRMT1 MFI in linear regression analysis.*P < 0.05; **P < 0.01; ***P < 0.001.

To further understand the relationships between PRMT1 and T cell function, we analyzed PRMT1 expression in cell populations with varying degrees of polyfunctionality. FACS-sorted CD8^+^ memory T cells were stimulated with anti-CD3/CD28 in the presence or absence of Wnt agonists for 7 days. By simultaneous detection of polyfunctional molecules and PRMT1 protein, we found that polyfunctionality positively correlated with PRMT1 expression in all treatment conditions (Figure 5, A and B). Comparing the MFI of PRMT1 from different cytokine-positive memory CD8^+^ T cells, the MFI of PRMT1 was highest among cells positive for IL-2, the first cytokine diminishing in the hierarchy of T cell exhaustion (11), with an average MFI of 3865, as compared with cells positive for IFN-γ and TNF-α, which had average PRMT1 MFIs of 2677 and 2857, respectively (Figure 5C). Furthermore, we found that PRMT1 expression was also correlated with cell proliferation (Supplemental Figure 6).

Since there are 3 dominant PRMT1 variants existing in T cells and the epitope of PRMT1 antibodies we used is not well characterized, we could not exclude the concern that the PRMT1 antibodies recognize a certain splicing variant that was reduced in the control group. To resolve this concern, we designed probes shared in all splicing variants to detect PRMT1 mRNA by PrimeFlow RNA Assay (Supplemental Table 1). Memory CD8^+^ T cells stimulated with or without Wnt agonist were analyzed for polyfunctionality and PRMT1 mRNA/protein levels. PrimeFlow RNA Assay showed that PRMT1 RNA expression was highly associated with PRMT1 protein expression and SKL2001 significantly increased both PRMT1 RNA and protein levels (Figure 5D). When we analyzed individual cytokine combinatorial populations, we found that SKL2001 globally enhanced the PRMT1 RNA and protein production in all 8 combinatorial groups (Figure 5E). We further calculated the polyfunctionality of the top 30% (PRMT1_hi_) and bottom 30% (PRMT1_lo_) of PRMT1-expressing cells and found PRMT1_hi_ cells demonstrated superior polyfunctionality in both DMSO and SKL2001 (Figure 5D) compared with PRMT1_lo_ cells. Nevertheless, the polyfunctionality in the SKL2001-treated PRMT1_lo_ group was still superior to that in the DMSO PRMT1_hi_ group (23% vs. 12%; Figure 5D). Comparing IFN-γ^+^, TNF-α^+^, and IL-2^+^ populations between PRMT1_hi_ and PRMT1_lo_, the percentage of cells expressing 3 cytokines was higher in PRMT1_hi_, albeit only IL-2 was statistically significant in both DMSO- and SKL2001-treated groups (Figure 5F). The enhancement of T cell polyfunctionality was most prominent in PRMT1_hi_ terminally differentiated CD8^+^ T cells (Supplemental Figure 7). Although data represented in Figure 5, B and D, showed that PRMT1 was positively associated with T cell polyfunctionality, different treatments partitioned the results into multiple groups, fragmenting the way in which data can be analyzed. To strengthen the cause-effect relationship between PRMT1 expression and polyfunctionality regardless of inducing factors, we calculated the polyfunctionality of subgroups from SKL2001-treated and nontreated memory CD8^+^ T cells stratified into multiple tiers based on their PRMT1 expression. The results demonstrated that T cell polyfunctionality is highly associated with PRMT1 expression (*R^2^* = 0.890) regardless of treatment (Figure 5G).

### PRMT1 epigenetically controls T cell polyfunctionality.

**Figure 6:**
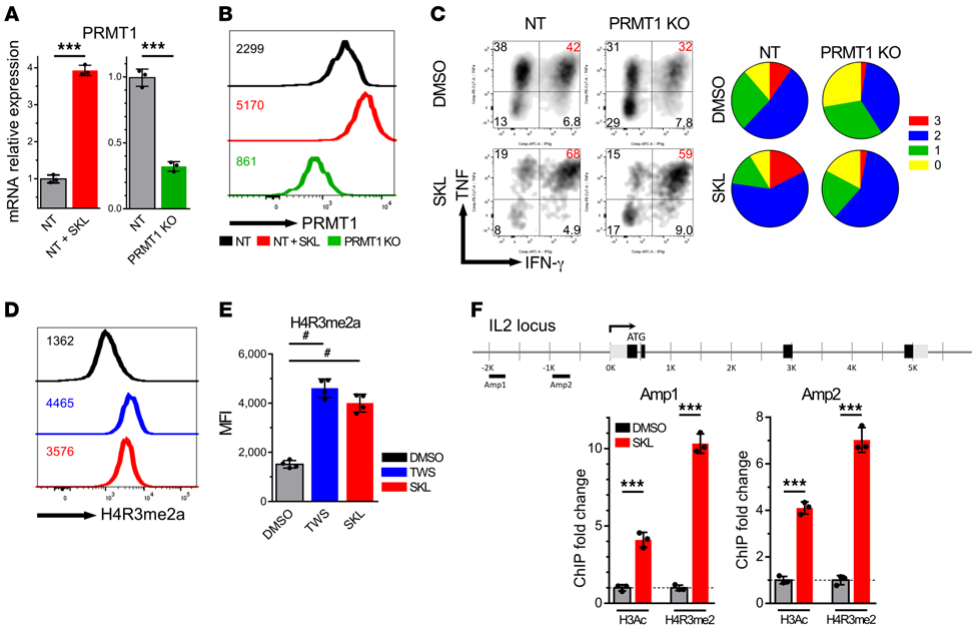
PRMT1 epigenetically controls CD8^+^ T cell polyfunctionality. (**A**) Memory CD8^+^ T cells were stimulated with CD3/CD28 in the presence or absence of SKL2001 for 1 day and transduced with either nontarget (NT) sequence virus or PRMT1 knockdown virus. On day 7, PRMT1 RNA expression was analyzed (*n =* 3). Mann-Whitney *U* test. (**B**) PRMT1 protein detection by flow cytometry in PRMT1 knockdown or nontarget virus in presence or absence of SKL2001. (**C**) Polyfunctionality profile of cells transduced with nontarget or PRMT1 knockdown virus in DMSO or SKL2001 treatment. 0, 1, 2, 3 are defined as the number of positive cytokines. (**D** and **E**) H4R3 dimethylation by PRMT1 was enhanced by Wnt agonists (*n =* 4). Dunn’s test for multiple comparisons. (**F**) ChIP assay of memory CD8^+^ cells stimulated with DMSO or SKL2001 for 7 days. ChIP assays were performed with antibodies to H3Ac and H4R3me2a. Each ChIP eluate was amplified by qPCR at the indicated regions of the IL-2 locus (*n =* 3). Mann-Whitney *U* test. ***P < 0.001. ^#^*P* < 0.05 by Dunn’s test.

Next, we investigated the mechanism by which PRMT1 improves T cell polyfunctionality. First, we engineered lentiviral particles containing shRNA-targeting PRMT1 to suppress its production. The shRNA was approximately 70% effective at suppressing PRMT1 mRNA (Figure 6A), while SKL2001 increased PRMT1 mRNA approximately 4-fold compared with control nontarget shRNA, consistent with the previous results (Figure 4E). The PRMT1 knockdown also led to the suppression of PRMT1 protein production (Figure 6B), as detected by flow cytometry (MFI, 2299 vs. 861). The polyfunctionality assay revealed that the PRMT1 knockdown T cells produced fewer cytokines than control virus–infected T cells and demonstrated decreased overall polyfunctionality. The PRMT1 knockdown virus also reversed the effect of SKL2001 on the enhancement of polyfunctionality (Figure 6C). The results indicated that PRMT1 is upstream of the polyfunctional response regulated by the Wnt pathway.

The effects of Wnt activation on T cell function were maintained after removal of the chemicals and after further cell division (Figure 2E); thus, we hypothesized an epigenetic mechanism is involved in the regulation of T cell polyfunctionality mediated by PRMTs. PRMTs catalyze arginine methylation, which is a common posttranslational modification (PTM) that plays a key role in the regulation of molecular and cellular responses, especially via histone methylation (38, 39). PRMT1 is responsible for asymmetric dimethylation of histone H4 at arginine 3 (H4R3me2a), which is associated with active transcriptional regulation (40, 41). We first used anti-H4R3me2a antibodies to evaluate overall histone methylation at the H4R3 residue in CD8^+^ T cells and found that Wnt agonists increased the total histone methylation (Figure 6, D and E). We hypothesized that PRMT1 directly methylates H4R3 in effector molecule loci, thus augmenting the accessibility of transcription factors and leading to superior polyfunctionality. We performed ChIP quantitative PCR (ChIP-qPCR) analysis to examine chromatin states in the promoter region of IL-2, which is the main determining function in polyfunctionality. We utilized PRMT1-mediated and well-known permissive histone marker antibodies anti-H4R3me2a and anti-H3Ac to pull down the modified chromatin from SKL2001-treated memory CD8^+^ T cells. Several amplicons specific for the IL-2, IFN-γ,and CD28 promoter regions were used to amplify the ChIP eluate (Figure 6F and Supplemental Figure 8). There was more than a 4-fold increase of H3 acetylation and a 7-fold increase of H4R3 dimethylation (normalized to DMSO-treated samples) in the amplicon regions of the IL-2 locus following SKL2001 treatment (Figure 6F). SKL2001 treatment also led to increased H3 acetylation and H4R3 dimethylation in the IFN-γ and CD28 loci (Supplemental Figure 8). These results indicated that PRMT1 regulates effector gene expression via methylation of H4R3 and thereby enhances T cell polyfunctionality.

### Wnt agonists prolong T cell survival in vivo and increase T cell polyfunctionality in CMV D+/R– LT recipients.

**Figure 7:**
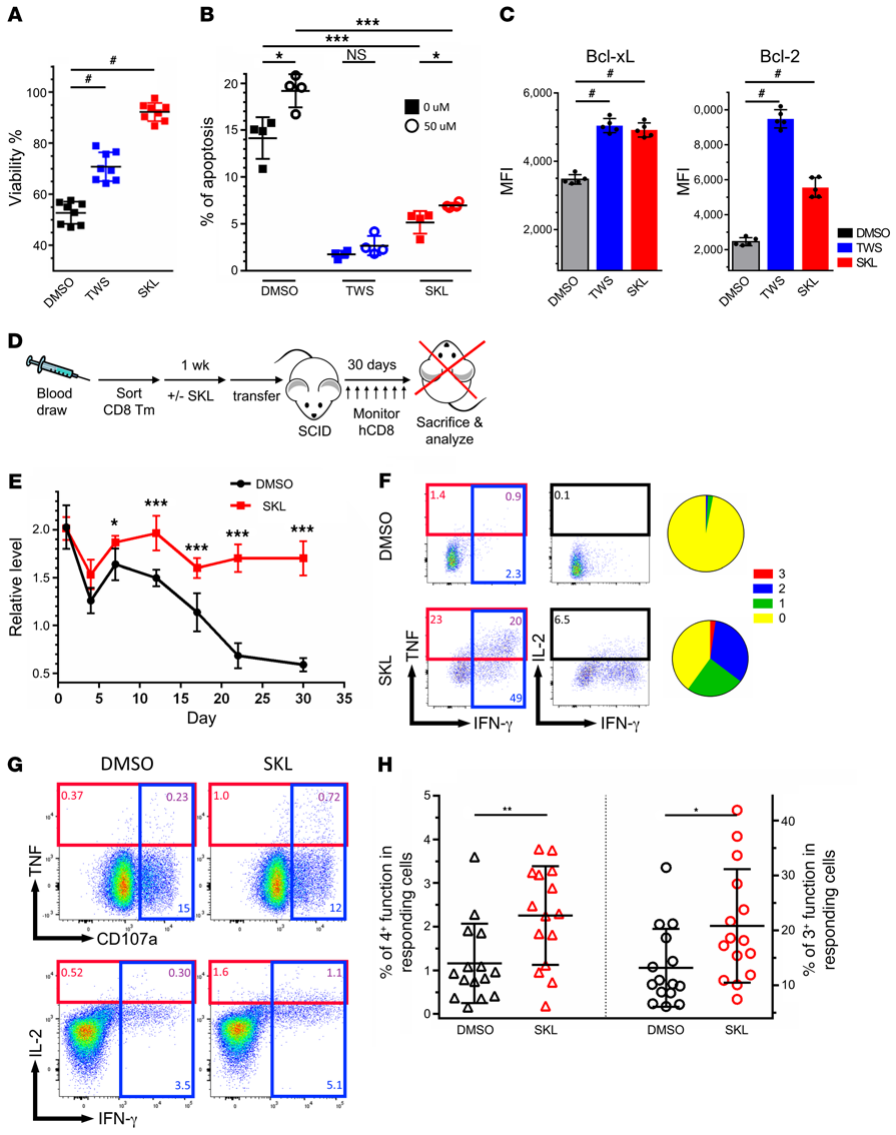
Wnt activation increases T cell survival and CMV-specific polyfunctionality in CMV D+/R– LT patients. (**A**) Human memory CD8^+^ T cells were stimulated with CD3/CD28 and treated with DMSO, TWS119, or SKL2001. On day 7, cells were replated at the same concentration in regular culture medium in the absence of further TCR stimulation and Wnt agonists for 7 days. Viability was analyzed on day 14 (*n =* 8). Dunn’s test for multiple comparisons. (**B**) Memory cells in different treatment conditions for 7 days were treated with 0 μM or 50 μM of cisplatin for 6 hours to induce apoptosis. Annexin V and 7-AAD were stained to analyze apoptotic cells. Percentages of Annexin V^+^/7-AAD^+^ in each condition were plotted (*n =* 4). (**C**) Antiapoptotic proteins Bcl-xL and Bcl-2 were analyzed by flow cytometry in memory CD8^+^ T cells with or without Wnt agonists for 7 days (*n =* 5). Dunn’s test for multiple comparisons. (**D**) Schematic showing the design of adoptive transfer experiment. Healthy donor memory CD8^+^ cells were sorted from PBMCs and treated with or without SKL2001 for 7 days; 5 × 10^6^ autologous CD4^+^ T cells and 2 × 10^6^ cultured CD8^+^ T cells were cotransferred into SCID mice (*n =* 5). CD8^+^ cells were monitored periodically. Thirty days after injection, T cells were harvested and a polyfunctionality assay was performed. (**E**) CD8^+^ T cell persistence was assessed over 1 month after adoptive transfer. (**F**) Polyfunctionality assay of transferred T cells on day 30. (**G**) PBMCs from CMV-infected (D+R–) LT recipients were isolated and stimulated with CMV pp65 peptide pool for 7 days. On day 7, PBMCs were restimulated with pp65 peptide pool for 8 hours. CD107a, TNF-α, IFN-γ, and IL-2 were analyzed for the polyfunctionality assay. (**H**) CMV-specific polyfunctionality was improved in the cells of 15 CMV D+/R– LT patients following DMSO or SKL2001 treatment (*n =* 15). Mann-Whitney *U* test. *P < 0.05; ***P < 0.001. ^#^*P* < 0.05 by Dunn’s test.

In the microarray analysis, we found that Wnt agonist–treated cells were enriched with stem cell–related genes. To test the survival potency of Wnt agonist–treated memory T cells for clinical applications, we studied the antiapoptotic features of the cells in vitro and the homeostatic maintenance of the cells in vivo. Memory CD8^+^ cells were stimulated in the presence or absence of Wnt agonists for 7 days, after which we redistributed equal numbers of cultured T cells without anti-CD3/CD28 stimulation for an additional 7 days. We found that Wnt-treated cells were more viable (Figure 7A) and expressed greater levels of the antiapoptotic molecules Bcl-xL and Bcl-2 (Figure 7C). Wnt agonists also contributed to the resistance to apoptosis in a cisplatin-induced apoptosis assay, as detected by annexin V and 7-AAD staining (Figure 7B). To evaluate T cell homeostasis in vivo, we transferred 2 × 10^6^ SKL2001-treated or nontreated human memory CD8^+^ T cells along with 5 × 10^6^ autologous CD4^+^ T cells into NOD SCID mice and monitored the dynamics of injected CD8^+^ T cells periodically (Figure 7D). The Wnt-treated human CD8^+^ T cells persisted longer and at higher levels than control CD8^+^ T cells, with 1.7-fold of cells remaining, as compared with just 0.6-fold of controls at 30 days (Figure 7E). Thirty days after injection, mice were sacrificed and the administered human T cells were isolated to perform a polyfunctionality assay. Following implantation, Wnt-treated CD8^+^ T cells were capable of producing more effector molecules compared with control cells, indicating the improved maintenance of polyfunctionality observed in vitro can be extended to applications in vivo (Figure 7F).

Finally, we utilized LT patient PBMCs to examine Wnt effects on CMV-specific T cell polyfunctionality. Polyfunctional immune response against CMV determines disease severity and frequency of recurrent episodes of CMV infection (42, 43). PBMCs were collected from CMV D+/R– LT recipients, who normally have difficulty controlling CMV reactivation and frequently suffer from repeated episodes of relapsing viremia, which are associated with allograft rejection and mortality (25). Detailed clinical information for these patients is listed in Methods. Patients’ PBMCs were acquired when CMV viremia was not detected and were stimulated with the CMV-pp65 peptide pool (0.4 μg/ml per peptide) for 7 days in the presence or absence of SKL2001. Cells were restimulated on day 7 to perform the polyfunctionality assay. As expected, SKL2001 treatment substantially enhanced the CMV pp65–specific polyfunctional responses in these high-risk LT patients (Figure 7, G and H). In conclusion, our findings shed light on the molecular mechanism by which the Wnt pathway epigenetically regulates T cell polyfunctionality by upregulating PRMT1 and suggest a potential treatment for enhancing immunotherapy against pathogens and cancer in clinical applications.

## Discussion

T cell polyfunctionality is a critical indicator for protective immunity against chronic viral infection and cancer. Ours and others’ previous research has found that the strength and duration of T cell activation are inversely correlated with polyfunctionality (5, 11), with excessive stimulation leading to impaired polyfunctionality and T cell exhaustion. It is known that many molecules, such as PD-1, T cell immunoglobulin and mucin domain 3 (TIM-3), CTLA-4, lymphocyte activation gene 3 (LAG-3), sprouty homolog 2 (SPRY2), eomesodermin (EOMES), and B lymphocyte–induced maturation protein 1 (BLIMP-1), regulate CD8^+^ T cell differentiation and exhaustion(5, 11, 12, 44), whereas CD62L, CCR7, CD127, costimulatory receptors, and T-bet are enriched in memory precursor or less-differentiated CD8^+^ T cells (11, 45).

In the past decade, Wnt signaling was identified as an important pathway to determining T cell stemness and differentiation (20, 21). In our study, we found that Wnt agonists TWS119 and SKL2001 elicit a robust polyfunctional immune response from memory CD8^+^ T cells. These findings do not contradict previous observations by Gattinoni et al. (31), as they investigated the effects of Wnt agonist on direct ex vivo induction of TSCM cells from TN cells, while here we focus on the effects of Wnt activation on antigen-experienced and memory CD8^+^ T cell proliferation and function. Importantly, the suppressive effect on memory T cell proliferation has not been seen in SKL2001-treated T cells, which may result from higher specificity of SKL2001 than TWS119 regarding Wnt pathway activation (32).

In T cell exhaustion, loss of individual effector molecules follows the hierarchical order of IL-2, TNF-α, IFN-γ, and proliferation during the establishment of chronic viral infection (11, 44). T cell function, particularly decreased IL-2 expression, highly correlates with cell proliferation. Our results demonstrate that Wnt activation enhanced polyfunctionality independently of cell proliferation. To study the long-term effects of Wnt agonists on T cell function, we designed a model to dissect the natural process of polyfunctionality loss in vitro. We found that the alteration of polyfunctionality regulated by Wnt pathway agonists can be preserved after drug removal, which is a crucial feature for clinical application and mechanistic studies. Clearly, Wnt activation alters intrinsic T cell mechanisms that maintain T cells in a highly functional state during T cell proliferation.

The induction of antigen-specific polyfunctional T cells is of pivotal importance for adoptive immunotherapy. We found that Wnt agonists enhance early memory CD8^+^ T cell polyfunctionality with all 3 different antigen-specific stimulations: moDCs, PBMCs, and aAPCs. The aAPC stimulation revealed that the enhancement of polyfunctionality is not dependent on the effects of Wnt signaling on antigen-presenting cells and provides a simple and feasible strategy to expand the superiority of antigen-specific T cells for therapy. In addition, we found that Wnt agonists can also reverse the inferior polyfunctionality of terminally differentiated, CMV pp65 (NLVPMVATV)–specific CD8^+^ T cells (Supplemental Figure 4). Nevertheless, in our system, we found that anti–PD-1 antibodies did not significantly increase T cell polyfunctionality (defined by the percentage of cells with multiple functions), although it upregulated individual effector molecule expression (defined by effector function MFI). This phenomenon may suggest that the clinical responses of PD-1 blockade are mostly determined by the number of preexisting functional T cells before checkpoint therapy. However, more evidence is required to support this speculation.

Previous studies have broadly reported that PRMTs are involved in multiple cellular processes, including subcellular localization, protein-protein interaction, signal transduction, cell cycle, transcription regulation, and chromatin remodeling (37, 39, 46). PRMT1 is the major enzyme in the human PRMT family, which mono- and asymmetrically dimethylates various protein substrates. PRMT1-deficient mice are embryonic lethal, demonstrating the importance of arginine methylation in developmental processes (46). Studies have shown that the alteration of PRMT1 function can modulate production of T cell cytokines, including IFN-γ, IL-2, IL-4, and IL-17, by mediating Vav1, NIP45, NFAT, STATs, or histone 4 arginine 3 asymmetric demethylation (H4R3me2a), which generally facilitates gene activation (40, 47). Methylation of Axin by PRMT1 can stabilize the destructive complex, which is associated with the downregulation of Wnt signaling. However, a recent study has shown that PRMT1 is required in the canonical Wnt pathway for the regulation of GSK3 phosphorylation (48). In our study, we tried to clarify the role of PRMT1 in memory T cell polyfunctionality. Given that polyfunctionality is an integrated index composed of multiple effector molecules, we clustered hundreds of cells in a group based on their PRMT1 expression followed by the polyfunctionality calculation for each group and discovered that T cell polyfunctionality is strongly correlated with PRMT1 expression. In our system, we did not observe suppression of cytokine production by PRMT inhibitors (data not shown), yet some groups have addressed this observation (49). The differences may result from the low specificity of the PRMT inhibitors or the experimental cells used (primary human CD8^+^ T cells vs. the Jurkat cell line). Most importantly, we show that viral transduction to knock down PRMT1 is capable of decreasing T cell function and reversing the effects of Wnt agonists on polyfunctionality. We also demonstrate that PRMT1 regulates effector molecule production through chromatin remodeling on the IL-2 locus.

In the manufacturing process of adoptive cell therapy, immune cells undergo multiple phases, including stimulation, selection, editing, and engineering. Manipulation of immune cells tends to render them fragile and vulnerable and reduces treatment efficacy. To examine cell contraction and homeostatic maintenance in host bodies after adoptive cell transfer, we injected SKL2001-treated human memory CD8^+^ T cells into SCID mice to monitor their persistence in vivo. SKL2001-treated cells not only persist more effectively than control cells, but also preserve their superior polyfunctionality. The observation validates our microarray analysis that Wnt activation in memory T cells preserves the “stemness” of expanded T cells.

A previous study (50) has shown that certain transplant patients harbor a restricted CMV-specific T cell response, especially loss of IL-2 secretion. These dysfunctional CMV-specific T cells contribute to susceptibility to clinical disease and represent a functional anergy state of T cells. While our results indicate an overall improvement in CMV-specific T cell response in unselected D+/R– transplant patients, further attention should be paid to investigating both the molecular and functional effects of Wnt agonists on the functional recovery of the dysfunctional cells in those patients.

In conclusion, this report demonstrates that Wnt agonists trigger the formation of long-lived CD8^+^ T cells with superior function via PRMT1 epigenetic modification at effector gene loci. The modulation of the Wnt pathway or PRMT1 expression could be a valuable approach to manipulating T cell polyfunctionality and immunological memory against chronic infectious diseases or malignancies.

## Methods

### Isolation of human PBMCs and CD14^+^ and CD8^+^ T cells.

PBMCs from 8 healthy HLA-A*0201^+^ donors and 15 CMV D+/R– LT patients were isolated by Ficoll-Paque PLUS gradient centrifugation (GE Healthcare). In some experiments, CD14^+^ and CD8^+^ T cells were isolated from PBMCs using the CD14-positive selection and CD8-negative selection kits, respectively, according to the manufacturer’s instructions (Miltenyi Biotec). For some experiments, naive and memory CD8^+^ T cells were purified by cell sorter (BD FACSAria III Cell Sorter) based on the surface markers CD8, CD45RA, CCR7 and CD62L.

### Generation of peptide-pulsed APCs/aAPCs and T cell expansion.

The generation of moDCs was achieved according to the protocol previously described (51). Briefly, CD14^+^ cells were cultured in complete RPMI 1640 medium supplemented with 5% autologous plasma, 100 ng/ml GM-CSF, and 50 ng/ml IL-4. After 6 days of culture, a maturation cocktail containing 10 ng/ml TNF-α, 10 ng/ml IL-1β, 1000 U/ml IL-6, and 1 μg/ml prostaglandin E2 was added for 1 day. MoDCs were harvested on day 7, and the maturation was confirmed by analysis of CD80, CD86, and HLA-DR. MoDCs were further pulsed with 1 μM influenza virus M1 (58–66; GILGFVFTL) for 2 hours at 37°C. After peptide loading, moDCs were extensively washed to remove free peptide. For the generation of aAPCs, M1 peptide–loaded HLA-A*02-Ig and anti-CD28 antibodies (clone 9.3) were biotinylated and coupled to 4.5 μm anti-biotin–coated microbeads (Miltenyi Biotec; ref. 34).

Three different methods were used to achieve antigen-specific expansion. In most antigen-specific expansion experiments, 3 million freshly purified CD8^+^ T cells were cocultured with 1 million peptide-pulsed moDCs in complete RPMI 1640 medium containing 5% autologous plasma, 2% T cell growth factor (51) with DMSO, or 3 μM TWS119 (Cayman Chemical) or 20 μM SKL2001 (Merck Millipore) for 7 days. In aAPC stimulation experiments, 3 million purified CD8^+^ T cells were cocultured with 3 million peptide-loaded aAPCs for 7 days. On day 7, T cells were harvested and counted, and T cells were replated at the same T cell/aAPC ratio if another round of stimulation was needed. In the experiments to dissect the synergistic effects of PD-1 blockade (10 μg/ml, clone EH12.2H7) and Wnt agonists, 5 × 10^5^ freshly isolated PBMCs were directly cultured in the medium containing 1 μM peptide in 1 well of a 96-well plate. For experiments in cells from CMV D+/R– LT recipients, 5 × 10^5^ thawed PBMCs were cultured in 1 well of a 96-well plate containing CMV pp65 peptide pool (PepMix JPT) at a concentration of 0.4 μg/ml per peptide for 7 days. Antigen specificity was assessed by HLA-A*02:01 Influenza-M1 tetramer (MBL International Corp.). For non–antigen-specific CD8^+^ T cell expansion, 10 μg/ml anti-CD3 (clone HIT3a) and 10 μg/ml anti-CD28 (clone CD28.2) were added for 2 hours at 37°C to generate precoated plates for T cell stimulation. Three million sorted CD8^+^ T cells were cultured on previously treated plates in the regular medium mentioned above with DMSO or 3 μM TWS119 or 40 μM SKL2001 for 7 days.

### Polyfunctionality profile measurement.

For antigen-specific stimulation experiments, 2 × 10^5^ CD8^+^ T cells were incubated with 2 × 10^5^ peptide-loaded HLA-A*02:01 T2 cells at 37°C in the presence of monensin (BD GolgiStop), brefeldin A (BD GolgiPlug), and anti-CD28/CD49d costimulatory reagent (BD Biosciences) for 8 hours. CD107a antibodies and tetramer were added at the beginning of the stimulation. T2 cells were pulsed with 1 μM peptide for 2 hours before use, and unpulsed (empty) T2 cells were used as background control. After 8 hours, cells were washed with FACS wash buffer followed by viability and surface staining at 4°C for 20 minutes. Cells were fixed and permeabilized by Cytofix/Cytoperm solution (BD Biosciences). After permeabilization, anti–IL-2, anti–TNF-α, and anti–IFN-γ antibodies were added and cells were incubated at 4°C for 90 minutes. For LT experiments and PBMC stimulation, CMV pp65 peptide pool and influenza M1 peptide, respectively, were added to restimulate T cells after 7 days of stimulation for cytokine production detection. For non–antigen-specific experiments, 3 × 10^5^ CD8^+^ T cells were incubated at 37°C in the presence of monensin, brefeldin A, 10 ng/ml PMA, and 1 μg/ml ionomycin for 6 hours followed by the same staining protocol mentioned above. After intracellular cytokine staining, cells were analyzed by flow cytometer BD LSR II. All cytokine combinations of different effector functions were performed using the Boolean combination gate by FlowJo software (version 10.1) (Tree Star). Exported results were further adjusted by background subtraction, and the pie charts were generated based on the summation of the same degree of polyfunctionality.

### Antibodies, tetramer, CTV, flow cytometry, and cell sorting.

We purchased fluorochrome-conjugated antibodies CD8–Brilliant Violet 605 (clone 301040, clone RPA-T8), CD4-PerCP/Cy5.5 (clone 344608, clone SK3), CD19-PerCP/Cy5.5 (clone 302230, clone HIB19), CD14-PerCP/Cy5.5 (clone 367110, clone 63D3), CCR7–Alexa Fluor 488 (clone 353206, clone G043H7), CD45RA–Alexa Fluor 647 (clone 304154, clone HI100), CD28–Alexa Fluor 488 (clone 302916, clone CD28.2), CD62L-PE (clone 304806, clone DREG-56), CD27-PE/Cy7 (clone 356412, clone M-T271), KLRG1–Alexa Fluor 647 (clone 367704, clone SA231A2), IFN-γ–Alexa Fluor 647 (clone 502516, clone 4S.B3), IL-2–Brilliant Violet 421 (clone 500328, clone MQ1-17H12), IL-2–PE (clone 500307, clone MQ1-17H12), TCF1–Alexa Fluor 647 (clone 655204, clone 7F11A10), Bcl-2–PE (clone 633508, clone BCL/10C4), and mouse NKp46–Alexa Fluor 647 (clone 137628, clone 29A1.4) from BioLegend; β-catenin–PE (clone 12-2567-42, clone 15B8), Eomes–eFluor 660 (clone 50-4877-42, clone WD1928), T-bet–PE (clone 12-5825-82, clone 4B10), goat anti-rabbit IgG-APC (clone A-10931), goat anti-rabbit IgG-PE (clone 31864), goat anti-mouse IgG-APC (clone A-865), and goat anti-mouse IgG-PE (clone P-852) from Invitrogen; and CD107a-FITC (clone 555800, clone H4A3), TNF-α–PE/Cy7 (clone 557647, clone MAb11), IFN-γ–BUV395 (clone 563563, clone B27) from BD Biosciences, and Bcl-xL-PE (clone 10030-09, clone 7B2.5) from Southern Biotech. Unconjugated antibodies PRMT1 (clone ab7027, clone MAT-B12), PRMT5 (clone ab10945, clone EPR5772), and H4R3me2a (clone ab194683) were purchased from Abcam. iTAg tetramer/PE–HLA-A*02:01 influenza-M1 (GILGFVFTL), iTAg tetramer/BV421 -HLA-A*02:01 influenza-M1, iTAg tetramer/BV421-HLA-A*02:01 CMV PP65 (NLVPMVATV), and iTAg tetramer/PE-HLA-A*02:01 CMV PP65 were purchased from MBL International Corp. to distinguish antigen-specific CD8^+^ T cells. We used FITC Conjugation Kit, PE/R–Phycoerythrin Conjugation Kit, and APC Conjugation Kit from Abcam to label the unconjugated antibodies. We performed surface staining at 4°C for 20 minutes. For intracellular staining, we used Cytofix/Cytoperm (BD) or Foxp3/Transcription Factor Staining Buffer (Invitrogen) to fix and permeabilize the cells. LIVE/DEAD Fixable Blue Dead Cell Stain Kit, for UV excitation and Fixable Viability Dye eFluor 780 were purchased from Invitrogen to identify viable cells. The CellTrace Violet Cell Proliferation Kit was purchased from Invitrogen to measure cell proliferation. the Annexin V–FITC Apoptosis Detection Kit with 7-AAD from BioLegend was used to detect apoptotic cells. All the procedures were performed following the manufacturer’s protocols. We carried out flow cytometry acquisition on LSR II (BD) equipped with 4 lasers and 10 PMT detectors. We analyzed the results and optimized tSNE distribution with FlowJo software (version 10.1) (Tree Star). We sorted CD8^+^ T cell subsets based on the surface staining using the FACSAria III Cell Sorter (BD).

### RNA detection by flow cytometry and qPCR.

The expression of PRMT1 mRNA was detected by the human PrimeFlow RNA Assay (probe design in Supplemental Table 1) from Invitrogen following the manufacturer’s instructions. Briefly, stimulated CD8^+^ T cells for PRMT1 expression and polyfunctionality assay were collected and stained with surface staining using anti-CD8 and dump detection reagents including anti-CD4, anti-CD19, and Fixable Viability Dye eFluor 780. Following cell-surface staining, cells were fixed and permeabilized by fixation and permeabilization buffer, followed by intracellular staining for 90 minutes at 4°C with anti–IL-2, anti–TNF-α, and anti–IFN-γ antibodies. Cells were further fixed with a second fixation buffer for 60 minutes at room temperature and then hybridized with human type 1 PRMT1 Alexa Fluor 647 RNA probe for 2 hours at 40°C. After multiple hybridization steps, the RNA probes were amplified and labeled. Cells were acquired on an LSR II flow cytometer (BD).

For qPCR to measure the mRNA, cells of interest were lysed in TRIzol reagent (Invitrogen), and total RNA was isolated by Direct-zol RNA Miniprep (Zymo Research). After cDNA conversion, the candidate genes were analyzed by real-time qPCR amplification on a 7900HT Fast Real-Time PCR System (Applied Biosystems) using the probes from TaqMan Assay Gene Expression (Thermo Fisher). 18S and ACTB were used as internal controls to adjust the expression of target genes.

### Lentivirus.

shRNA (sequences are listed in Supplemental Table 1) targeting all variants of human PRMT1 gene or scramble sequence in the pLKO.1 pure plasmid was purchased from the Johns Hopkins University High Throughput Biology Center (HiT). Viral particles were produced by delivering 7.5 μg shRNA-pLKO.1 plasmid, 5.6 μg pCMV-dR8.91 (Delta 8.9) plasmid, and 1.9 μg pCMV-VSV-G plasmid mixed with 45 μl TransIT-2020 (Mirus) into HEK293T cells (75% confluency in 10 cm dish). Viral particles were concentrated by Lenti-X Concentrator (Clontech) following the manufacturer’s instructions. Transduction of primary CD8^+^ T cells was performed 24 hours after anti-CD3/CD28 stimulation and drug treatment by spinoculation at 300 *g* for 60 minutes at 32°C in the presence of 8 μg/ml polybrene (Sigma-Aldrich). Twenty-four hours after transduction, puromycin was added at a final concentration of 1.5 μg/ml to select virus-infected T cells. Six days after viral transduction, CD8^+^ cells were harvested to evaluate the knockdown efficiency and polyfunctionality by qPCR and flow cytometry.

### Cytotoxicity and viability assay.

Antigen-specific CD8^+^ cells were stimulated for 7 days by peptide-pulsed moDCs mentioned above. Target T2 cells (expressing HLA-A2) were divided into 2 populations, labeled with 2 different intensities of CTV. CTV_hi_ cells were pulsed with cognate peptides, while the CTV_lo_ portion remained unpulsed. CTV_hi_ and CTV_lo_ cells were mixed at a 1:1 ratio before adding to effector cells. After harvesting the antigen-specific T cells, target T2 cells containing the 2 groups were added at various effector/target ratios and incubated for 8 hours. Cell density was adjusted to 10,000 cells/well in a 96-well plate. The results were obtained by flow cytometry, and the specific killing ability was calculated by the ratio of CTV_hi_ to CTV_lo_ cells. Fixable Viability Dye eFluor 780 was used for viability assay, and Apoptosis Detection Kit (BioLegend) was used to measure cisplatin-induced apoptosis.

### Microarray and RNA-Seq analysis.

Sorted human memory CD8^+^ T cells from 3 donors were stimulated with anti-CD3/CD28 antibodies for 7 days in the presence of TWS119 or DMSO. More than 1 million cells were harvested and lysed in TRIzol reagent. RNA extraction was conducted using the PureLink RNA Mini Kit (Invitrogen). For RNA-Seq, RNA of each sample was qualified and quantified by Bioanalyzer (Agilent Technologies) and NanoDrop (Thermo Fisher), respectively. At least 500 ng total RNA of each sample was used to establish a library. mRNA was enriched by the NEBNext Poly(A) mRNA Magnetic Isolation Module (New England Biolabs). After reverse transcription, cDNA was amplified by P5 and P7 primers to accommodate the sequencer platform (Illumina Novaseq 6000). For microarray assay, cDNA of each sample was applied to single-colored Human Gene Expression 8× 60K Oligo Microarrays (Agilent Technologies) to measure gene expression.

Partek Genomics Suite was used to normalize gene expression between different samples and to analyze the expression changes in different treatments. Genes with a fold change greater than 1.5 and a *P* value of less than 0.05 were listed for further investigation. Differentially expressed genes were annotated and functionally grouped by DAVID (version 6.7), provided by the NIH (https://david.ncifcrf.gov/). Functional annotation and pathway analysis using GO were also performed on the DAVID platform. The complete microarray data set was deposited in the NCBI’s Gene Expression Omnibus (GEO GSE59400).

We performed GSEA to identify gene sets that were enriched in either TWS119-treated or control CD8^+^ cells. Data underwent log_2_ transformation prior to GSEA. Maximum expression ratio was chosen for GSEA calculation if multiple probes hybridized 1 gene target. We calculated the running sum of the statistics for each gene set. The test statistic was the maximum of the running run (enrichment score), and permutation tests were performed to evaluate the empirical *P* value of the enrichment.

### ChIP experiment.

5 × 10^6^ SKL2001 treated or control memory CD8^+^ T cells were harvested after 7 days of activation. ChIP analysis was performed using the Zymo-Spin ChIP Kit (D5210, Zymo Research) following the manufacturer’s instructions. Briefly, 4% of paraformaldehyde was added to fix the cells, and glycine was added afterward to cease the crosslink reaction. Cell pellets were suspended in Chromatin Shearing Buffer with protease inhibitor and fragmented by sonicator (Branson). The ChIP antibodies, anti-H3Ac (Millipore), anti-H4R3me2a (Abcam), and isotype control (BioLegend) were added into the sheared chromatin. ZymoMag Protein A Beads were incubated with ChIP reaction for 1 hour at 4°C. After multiple wash steps, protein A bead/antibody/chromatin complexes were resuspended in chromatin elution buffer. NaCl was added to the solution to dissociate protein A beads and chromatin complex. Proteinase K was added into ChIP DNA solution to degrade protein. Zymo-Spin IC column was used to isolate the ChIP DNA, and ChIP DNA was eluted by DNA elution buffer. Real-time qPCR amplification using SYBR Green PCR Master Mix (Applied Biosystems) was performed with 2 μl of immunoprecipitated DNA. The following primer sets were used: amplicon 1 on IL2 gene: sense 5′-TGTGGGGGCTTTTCCAATGA-3′, anti-sense 5′-TGTCAGACGTGAGAAAGCGA-3′; amplicon 2 on IL2 gene: sense 5′-ACAGTGCACAAACTGGCTGA-3′, anti-sense 5′-CCTGCCTGTACACTGTTCTCT-3′; amplicon 1 on IFNG gene: sense 5′-AAGCCTACGGTGCATCTCAAT-3′, anti-sense 5′-GGAGGGCTTTTTGTGCCATC-3′; amplicon 2 on IFNG gene: sense 5′-TATGCCCACCTGTGCCATTC-3′, anti-sense 5′-CTTGTTCCCAACCACAAGCAA-3′; amplicon 3 on IFNG gene: sense 5′-CTTGTTCCCAACCACAAGCAA-3′, anti-sense 5′-CAAGCTGATCAGGTCCAAAGGA-3′; amplicon 1 on CD28 gene: sense 5′-CTCCAAAGGACCAAAAGATGTGT-3′, anti-sense 5′-GAGGAGGCAGGCTTCATGTC-3′; and amplicon 2 on CD28 gene: sense 5′-GCTGCTTGCACGTAGAATGG-3′, anti-sense 5′-ATGGGGACAGGTTGTGTCAA-3′.

### Mouse T cell transplant persistent assay.

2 × 10^7^ Memory CD8^+^ T cells and 4 × 10^7^ total CD4^+^ cells were isolated from healthy donors by BD FACSAria Cell Sorter. CD8^+^ cells were divided into control and SKL2001-treated groups cultured for 7 days, while CD4^+^ T cells were stimulated by anti-CD3/CD28 antibodies. 2 × 10^6^ Cultured human CD8^+^ T cells and 5 × 10^6^ autologous CD4^+^ cells were adoptively transferred into each NOD SCID mouse (The Jackson Laboratory). At least 5 replicate mice received the same conditioned CD8^+^ T cells to avoid biological and technical variability. Tail blood was collected from recipient mice every 4 or 5 days after adoptive transfer to monitor injected human CD8^+^ T cell numbers. Mice were sacrificed 30 days after adoptive transfer and analyzed for the polyfunctionality of injected human CD8^+^ T cells from 2 groups.

### Statistics.

The statistics of polyfunctionality pie charts were performed by SPICE provided by the National Institute of Allergy and Infectious Diseases (NIAID). Other statistical analyses were performed by GraphPad Prism (version 7.) The nonparametric Wilcoxon’s matched-pairs signed-rank test was used on 2 related sample groups, and the Mann-Whitney *U* test was used on 2 independent sample groups. *P* values of less than 0.05 were considered statistically significant and are labeled with single asterisks, while *P* values of more than 0.05 are labeled NS. *P* values of less than 0.01 and 0.001 were labeled with double asterisks and triple asterisks, respectively. In cases of multiple groups comparisons, Dunnett’s test was used to correct the type I error resulting from the accumulation of numerous comparisons. Every experimental group was compared with a control group and α was set as 0.05. Mean of experimental groups beyond the confidence limits was considered statistically significant and labeled with hatch marks or with NS when difference was within the confidence interval.

### Study approval.

All healthy donor experiments were approved by the Research Ethics Committee of the Johns Hopkins University (NA_00027947 and MO17M77 for mice), and written informed consent was obtained from all human study participants. The LT patients receiving immunosuppressants and antiviral drugs were recruited from Johns Hopkins University. LT recipients in this cohort had undergone transplantation between 2004 and 2012 and were all CMV D+/R–. PBMC samples were obtained at a median of 26 months after transplant (15–118 months). During the era in which these samples were collected, CMV prophylaxis was administered as intravenous ganciclovir for 3 months for CMV D+/R– LT recipients. In this cohort, all but one patient had experienced previous CMV DNAemia, with 4 patients having had previous CMV end-organ disease (3 with CMV pneumonitis, 1 with CMV pneumonitis and colitis), but these episodes were remote, having occurred at a median of 23 months before the samples were obtained (7–113 months). No patient had active CMV DNAemia at the time of PBMC sampling. In terms of immunosuppression, 2 patients had received basiliximab for induction and the others had received no induction. Maintenance immunosuppression was prednisone, tacrolimus, and mycophenolate mofetil, except in 3 patients who received everolimus in place of mycophenolate. No patient in this cohort has had biopsy-proven rejection or intensification of immunosuppression within the 3 months prior to PBMC sampling. Clinical and biologic data for human LT recipients was obtained under the auspices of IRB00138643.

## Author contributions

BYS, YLC, and JPS conceived the study. BYS and YLC performed experiments and analyzed the data. QK assisted PRMT1 knockdown experiments. YHL conducted the ChIP experiment. PDS, SP, KW, and RKA participated in LT patient recruitment and experiments. HRC and HH analyzed the microarray and RNA-Seq data. BYS and YLC wrote the manuscript. JGB, YLC, RKA, and JPS revised the manuscript. JPS provided financial support and supervised the work.

## Supplementary Material

Supplemental data

## Version 1 01/18/2022

Electronic publication
