# Supplementary material for: Wnt activation promotes memory T cell polyfunctionality via epigenetic regulator PRMT1
Source: J Clin Invest. 2022 Jan 18;132(2):e140508. doi: 10.1172/JCI140508 (PMC8759796; doi:10.1172/JCI140508)
Supplement: Supplemental data [file jci-132-140508-s041.pdf]

Supplemental Information of Manuscript

Wnt Activation-Induced PRMT1 Epigenetically Controls Memory T cell Polyfunctionality

Bo-Yi Sung[1,2,3], Yi-Hsin Lin[3,4], Qiongman Kong[1], Pali D. Shah[5], Joan Glick Bieler[1,2], Scott Palmer[6], Kent Weinhold[7], Hong-Ru Chang[3], Hailiang Huang[8], Robin K. Avery[9], Jonathan P. Schneck*[1,2], Yen-Ling Chiu*[1,10,11,12]

[1]Institute of Cell Engineering, School of Medicine, Johns Hopkins University, Baltimore, Maryland, USA

[2]Department of Pathology, School of Medicine, Johns Hopkins University, Baltimore, Maryland, USA

[3]Department of Microbiology and Immunology, National Defense Medical Center, Taipei, Taiwan

[4]Department of Obstetrics and Gynecology, Tri-Service General Hospital, National Defense Medical Center, Taipei, Taiwan

[5]Division of Pulmonary and Critical Care Medicine, Department of Medicine, School of Medicine, Johns Hopkins University, Baltimore, Maryland, USA

[6]Department of Medicine, Duke University Medical Center, Durham, North Carolina, USA

[7]Department of Surgery, and Department of Immunology, Duke University Medical Center, Durham, North Carolina, USA

[8]Analytic and Translational Genetics Unit, Massachusetts General Hospital, Boston, Massachusetts, USA

[9]Division of Infectious Diseases, Department of Medicine, School of Medicine, Johns Hopkins University, Baltimore, Maryland, USA

[10]Graduate Institute of Medicine and Graduate Program in Biomedical Informatics, Yuan Ze University, Taoyuan, Taiwan

[11]Department of Medical Research, Far Eastern Memorial Hospital, Taipei, Taiwan

[12]Graduate Institute of Clinical Medicine, National Taiwan University College of Medicine, Taipei, Taiwan

*Correspondence: Dr. Jonathan P. Schneck and Dr. Yen-Ling Chiu

Supplemental Figure 1. Memory CD8+ T cell cytokine production with Wnt agonists

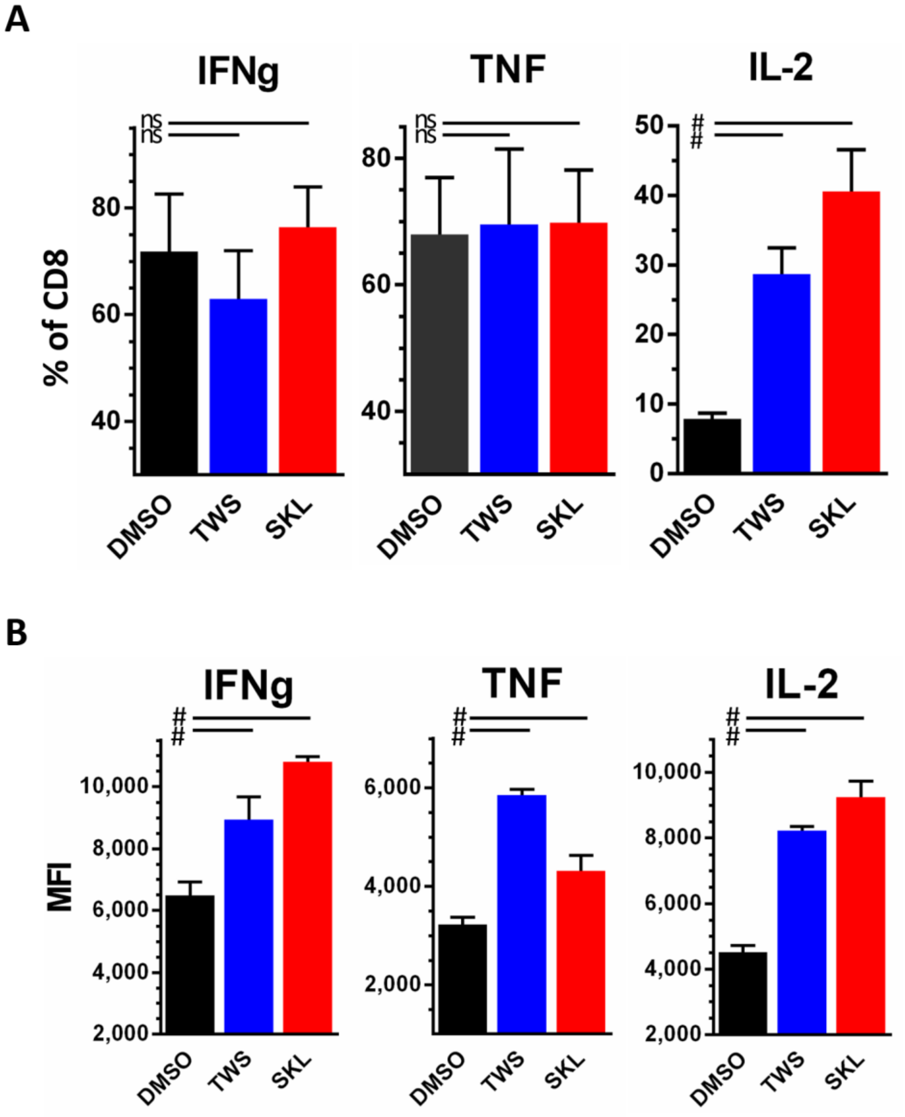

Memory CD8+ T cells stimulated for 7days in different treatments were analyzed for cytokine production. A. Percentages of IFN-γ, TNF-α, and IL-2 producing cells in the total memory CD8+ cell population (n=5). B. MFI of each cytokine in cytokine-producing cells (n=5).

Supplemental Figure 2. The polyfunctionality of naïve and memory CD8+ cells in the presence or absence of TWS119

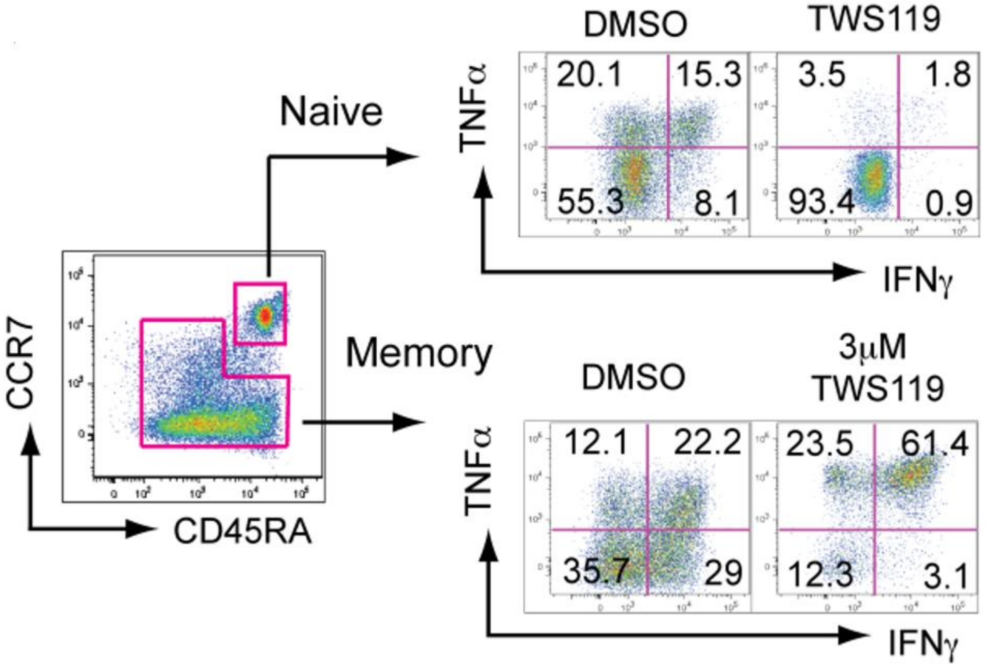

Naïve and memory T cells were sorted based on CD45RA and CCR7 expression, then activated with CD3/CD28 antibodies in the presence or absence of TWS119 (3μM). On day 7, cells were stimulated with PMA/ionomycin and analyzed cytokine expression.

Supplemental Figure 3. Comparison of CCR7 and CD62L defined naïve and memory CD8+ T cells

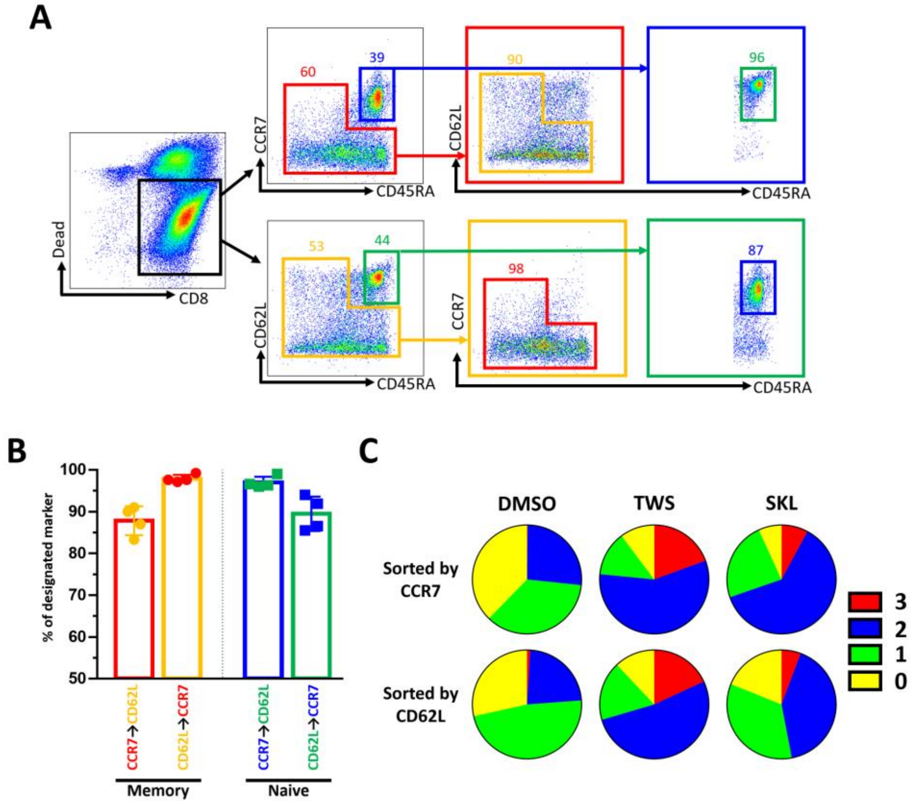

A. Naïve and memory CD8+ T cells were defined by CCR7/CD45RA and CD62L/CD45RA in direct *ex vivo* samples. In the top row, naïve CD8+ T cells were gated by $CD45RA^{hi}/CCR7^{hi}$ (blue region) and memory T cells were gated by non-$CD45RA^{hi}/CCR7^{hi}$ (red region). Naïve and memory CD8+ T cells defined by CCR7 were further analyzed by CD62L and CD45RA. In the bottom row, naïve and memory CD8+ T cells were first gated by $CD45RA^{hi}/CD62L^{hi}$ and non-$CD45RA^{hi}/CD62L^{hi}$ (green and orange regions), respectively. Naïve and memory CD8+ T cells were further analyzed by CCR7 and CD45RA, respectively.
B. A comparison of CCR7 versus CD62L to delineate naïve and memory T cells (n=4).
C. CD8+ memory T cells sorted based on CCR7/CD45RA (top row) or CD62L/CD45RA (bottom row) were stimulated and treated with or without Wnt agonists for 7 days. T cell polyfunctionality were presented by pie chart, in which different color of slices indicate various T cell function. The enhancement of T cell polyfunctionality induced by Wnt agonists were similar in CCR7 or CD62L defined memory CD8+ T cells.

Supplemental Figure 4. TWS119 and SKL2001 increase pp65-specific CD8+ T cell polyfunctionality stimulated by moDCs.

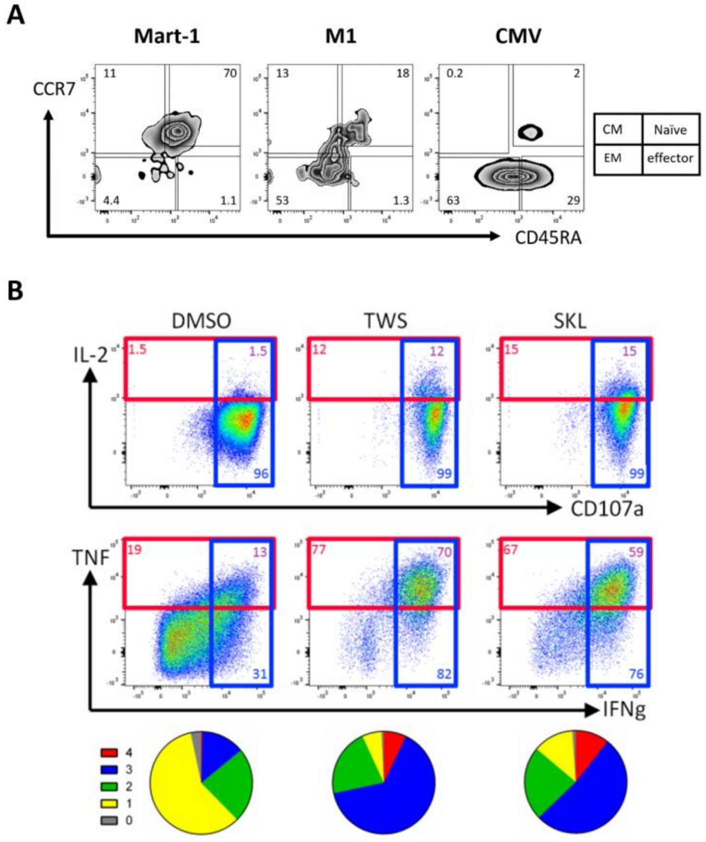

A. Direct *ex vivo* analysis of different antigen-specific CD8+ T cells differentiation status from HLA-A*0201+ donors. CD8+ T cells stained with Mart-1 (melanoma antigen recognized by T cells 1 or melan-A), M1 (influenza virus) and pp65 (cytomegalovirus, CMV) tetramers. Differentiation status were defined by CD45RA and CCR7 surface markers. Naïve cells (UR quadrant) are CD45RA+CCR7+; central memory (CM, UL quadrant) cells are CD45RA–CCR7+; effector memory (EM, LL quadrant) cells are CD45RA–CCR7–, and effector (T_{EMRA}, LR quadrant) cells are CD45RA+CCR7–. CMV-specific CD8+ cells consist of terminal differentiated cells, namely EM and effector T cells.

B. Polyfunctionality assay in CMV pp65 (NLVPMVATV) specific CD8+ cells after tetramer gating. Wnt agonists were able to increase polyfunctionality in terminal differentiated pp65-specific CD8+ T cells.

Supplemental Figure 5. RNA sequence revealed that Wnt agonists upregulate stem cell related genes and suppress exhaustion genes

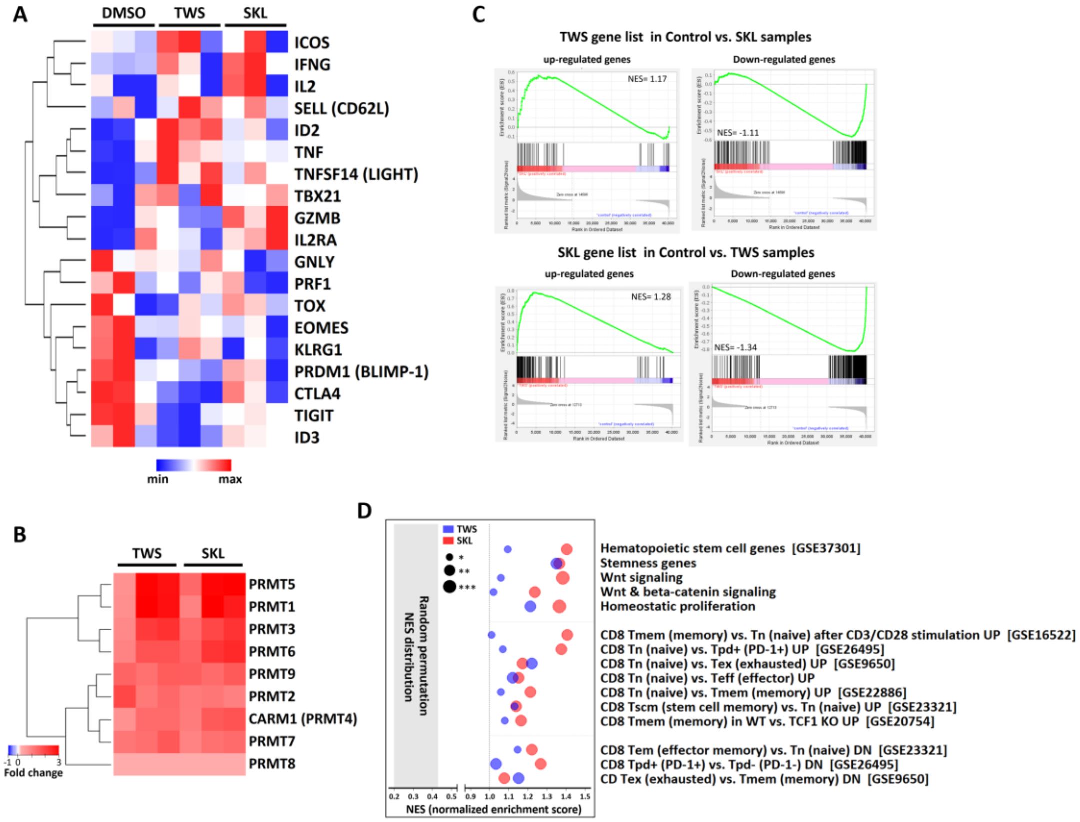

Memory CD8+ T cells were sorted from 3 donors and cultured in presence or absence of Wnt agonists for 7 days. Cells were harvested and RNA expression was investigated by RNA sequence.

A. Heat map demonstrated genes related to effector function, costimulatory molecules exhaustion and stemness.

B. Fold change of protein arginine methyltransferases (PRMTs) family under TWS119 and SKL2001 treatments.

C. Gene set enrichment analysis (GSEA) illustrates that the gene signatures are highly correlated in TWS119 and SKL2001 treated samples. In the top row, gene lists were extracted from TWS119 treated cells (FC>±2, p-value<0.05) and GSEA was depicted by SKL2001/DMSO comparison. Inversely, GSEA was depicted based on SKL2001 mediated genes by TWS119/DMSO comparison in the bottom row. The left panels are gene lists upregulated and the right panels are gene lists downregulated after treating with Wnt agonists.

D. Multiple gene sets were evaluated by GSEA in TWS119 and SKL2001 samples. Stemness and Wnt signaling gene sets were upregulated, whereas exhaustion gene sets were negatively correlated.

Supplemental Figure 6. PRMT1 is upregulated in divided CD8+ cells

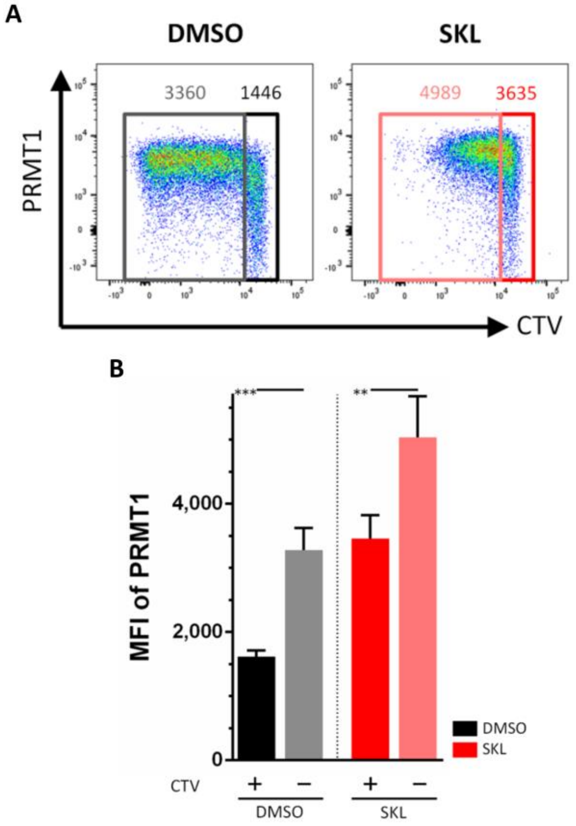

A. Memory CD8+ cells stained with CTV on day 0 and stimulated with CD3/CD28 for 7 days. Dot plots demonstrated the correlation between cell proliferation and PRMT1 expression. Numbers listed above gating were PRMT1 MFI of corresponding cells.
B. Bar graph summarized the MFI of PRMT1 in different populations. CTV+ indicated non-dividing cells and CTV− indicated dividing cells (n=5).

Supplemental Figure 7. Polyfunctionality profile and PRMT1 expression between different CD8+ T cell subsets

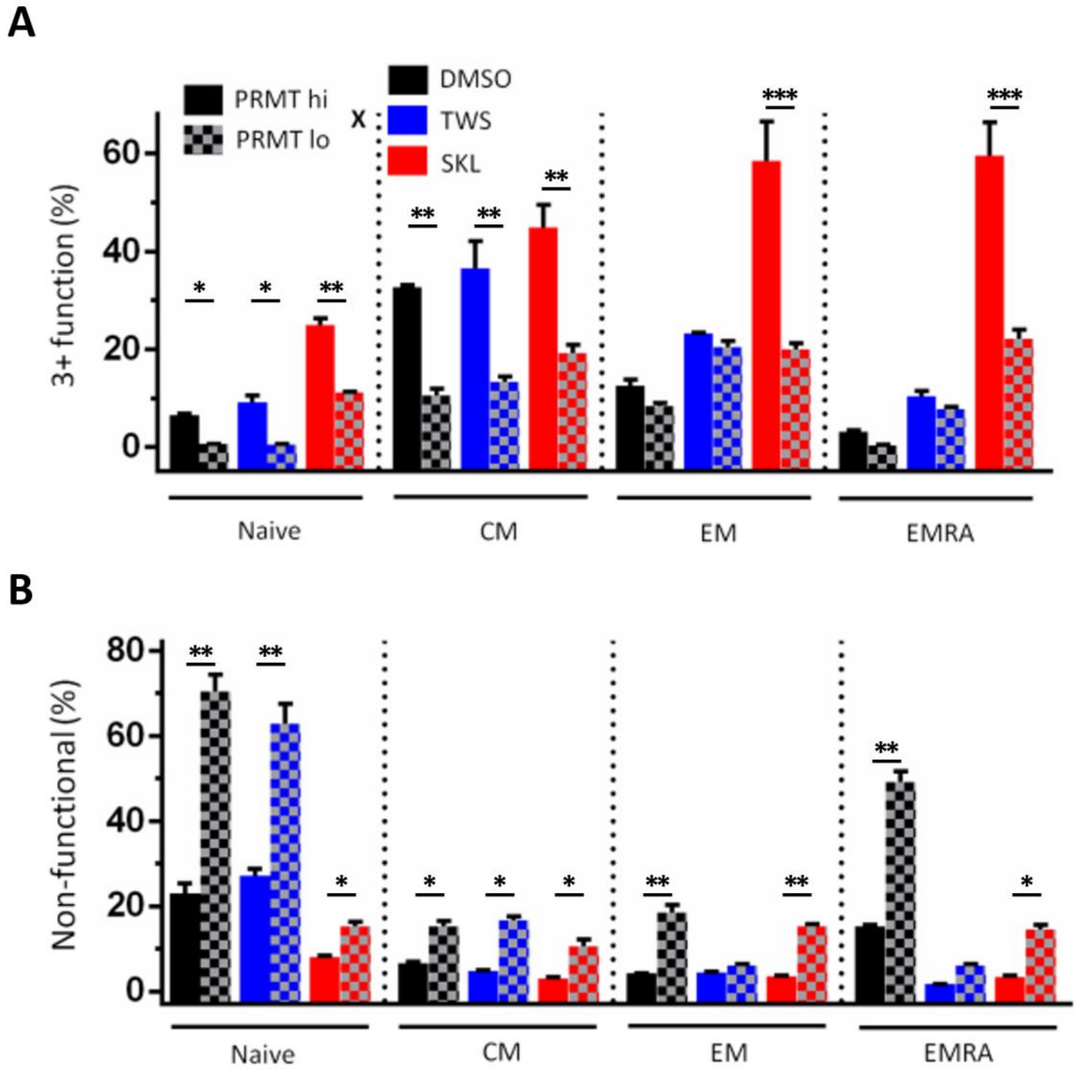

A. FACS-sorted Naïve (CD45RA+CCR7+), central memory (CM, CD45RA–CCR7+), effector memory (EM, CD45RA–CCR7–) and terminal effector (EMRA, CD45RA+CCR7–) CD8+ cells were stimulated by CD3/CD28 for 7 days in indicated treatment. Polyfunctionality assay combined with PRMT1 protein/mRNA staining was performed. Percentage of 3+ function (full functional) cells are shown for different precursor cells treated with TWS119 and SKL2001. PRMT1 high shown in filled or low in grid columns and DMSO, TWS119 or SKL2001 shown in black, blue or red, respectively.

B. Percentage of non-functional (dysfunctional or exhausted) cells were plotted from different precursor cells under indicated conditions. In central memory T cells, PRMT1 expression can clearly distinguish T cell polyfunctionality amongst the three conditions. The differences in polyfunctionality can be differentiated by PRMT1 expression among four precursor populations in SKL2001 treatment.

Supplemental Figure 8. ChIP assays displayed that Wnt activation enriched active histone modifications of IFNG and CD28 loci

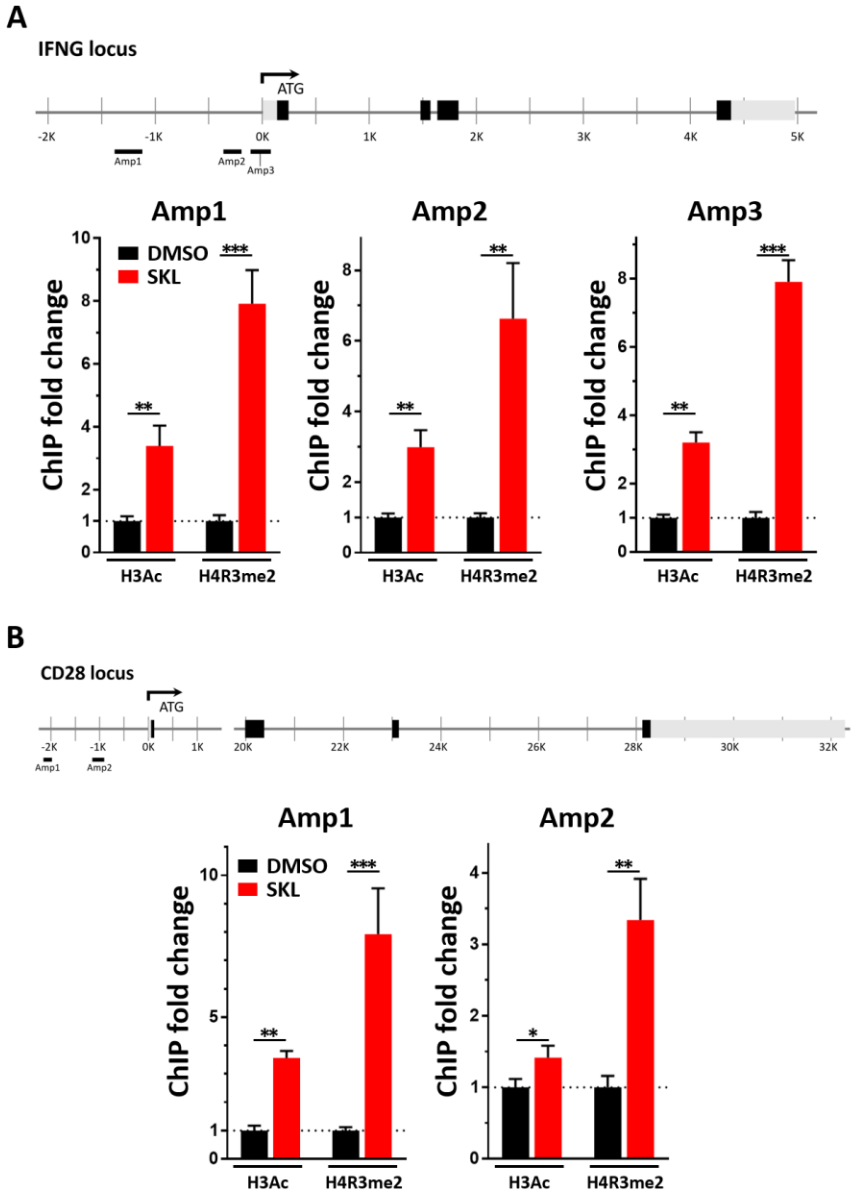

A. DMSO and SKL2001 treated chromatin was pulled down by H3Ac and H4R3me2a antibodies and 3 amplicons of the IFNG loci were evaluated by qPCR.
B. 2 amplicons of the CD28 loci were amplified by qPCR from the elution of ChIP assay. All ChIP fold change was normalized to the corresponding amplicon and histone marker of DMSO control.

Supplemental Table 1. PrimeFlow RNA probe design and shRNA knockdown sequences

Human PRMT1 variant1 mRNA

attgccaatgaaatcttccagcggggtcgcggtaggcgggccgtggaccctctggtataaggcggtcccggggagtgagg
agaaaggggggtcttggcggccggaggaggagtaggtgcgggtgaag**ATG**GCGGCAGCCGAGGCCG
CGAACTGCATCATGGAGAATTTGTAGCCACCTTGGCTAATGGGATGAGCCT
CCAGCCGCCTCTTGAAGAAGTGTCCTGTGGCCAGGCGGAAAGCAGTGAGAA
GCCCAACGCTGAGGACATGACATCCAAAGATTACTACTTTGACTCCTACGCA
CACTTTGGCATCCACGAGGAGATGCTGAAGGACGAGGTGCGCACCCTCACT
TACCGCAACTCCATGTTTCATAACCGGCACCTCTTCAAGGACAAGGTGGTGC
TGGACGTCGGCTCGGGCACCGGCATCCTCTGCATGTTTGCTGCCAAGGCCGG
GGCCCGCAAGGTCATCGGGATCGAGTGTTCCAGTATCTCTGATTATGCGGTG
AAGATCGTCAAAGCCAACAAGTTAGACCACGTGGTGACCATCATCAAGGGG
AAGGTGGAGGAGGTGGAGCTCCCAGTGGAGAAGGTGGACATCATCATCAGC
GAGTGGATGGGCTACTGCCTCTTCTACGAGTCCATGCTCAACACCGTGCTCT
ATGCCCGGGACAAGTGGCTGGCGCCCGATGGCCTCATCTTCCCAGACCGGGC
CACGCTGTATGTGACGGCCATCGAGGACCGGCAGTACAAAGACTACAAGATC
CACTGGTGGGAGAACGTGTATGGCTTCGACATGTCTTGCATCAAAGATGTGG
CCATTAAGGAGCCCCTAGTGGATGTCGTGGACCCCAAACAGCTGGTCACCAA
CGCCTGCCTCATAAAGGAGGTGGACATCTATACCGTCAAGGTGGAAGACCTG
ACCTTCACCTCCCCGTTCTGCCTGCAAGTGAAGCGGAATGACTACGTGCACG
CCCTGGTGGCCTACTTCAACATCGAGTTCACACGCTGCCACAAGAGGACCG
GCTTCTCCACCAGCCCCGAGTCCCCGTACACGCACTGGAAGCAGACGGTGT
TCTACATGGAGGACTACCTGACCGTGAAGACGGGCGAGGAGATCTTCGGCA
CCATCGGCATGCGGCCCAACGCCAAGAACAACCGGGACCTGGACTTCACCA
TCGACCTGGACTTCAAGGGCCAGCTGTGCGAGCTGTCCTGCTCCACCGACTA
CCGGATGCGC**TGA**ggcccggctctcccgccctgcacgagcccaggggctgagcgttcctaggcggtttcgggg
ctccccctteetctccctccctcccgcagaagggggttttaggggcctgggctgggggatggggagggcacatcgtgactg
tgtttttcataacttatgtttttatatggttgcatttacgccaataaatcctcagctggggtc

---

lowercase: untranslated region
UPPERCASE: CODING SEQUENCE
blocking labels (BL)
label extenders (LE)
shRNA target sites
translational start and stop sites
black regions are unique sequence in variant 1 (absent in variant 3 or 4)
